# Music-Evoked Nostalgia and Wellbeing During the United Kingdom COVID-19 Pandemic: Content, Subjective Effects, and Function

**DOI:** 10.3389/fpsyg.2021.647891

**Published:** 2021-03-22

**Authors:** Hannah Gibbs, Hauke Egermann

**Affiliations:** York Music Psychology Group, Department of Music, University of York, York, United Kingdom

**Keywords:** nostalgia, COVID-19, listening, music, wellbeing, emotion, regulation, lockdown

## Abstract

Nostalgic music is defined as that which evokes feelings of nostalgia through reminders of certain periods of life, places or people. Feelings of nostalgia are said to occur during times of hardship and difficult transitionary periods, such as the first COVID-19 lockdown in the United Kingdom in 2020. Here, the reassurance of the past might have held certainty that could sustain a sense of meaning and purpose in life and influence wellbeing. The aims of the presented study were to explore the nature of music-induced nostalgia during the lockdown, by analysing participants’ narratives conjured by the music and their emotional responses to them, and to determinethe extent that using nostalgic music listening as an emotion regulation strategy had an impact on wellbeing. Data was collected by means of an online questionnaire, which retrospectively investigated nostalgic music during the lockdown. Participants listened to a self-selected piece of music that they had listened to 3 months prior whichinduced feelings of nostalgia, reported their resulting emotion and the content of memories associated with their nostalgia, and completed a questionnaire rating their experienced effect of nostalgia in relation to their piece of music. Following this, we investigated the functions that nostalgic music tends to have in regulating emotions through means of a pre-validated scale. 570 participants (34% identified as male) were recruited (age years *M* = 44, *SD* = 16). Concurrent with existing research, the findings suggest that there are significant differences in the affective and narrative content of nostalgicmusic listening in relation to which emotion regulation strategy was used, and that employing nostalgic music listening as a form of approaching difficult emotions can have a positive impact on wellbeing.

## Introduction

Amidst the height of the COVID-19 crisis in the United Kingdom, day to day normality was impeded as a result of the enforcement of social distancing measures. The restrictions were enforced in England from March 23, 2020 (Prime Minister’s Statement on Coronavirus (COVID-19): 23 March 2020 - GOV.UK, 2020), when Prime Minister Boris Johnson announced that the public must stay at home unless shopping for essential items, exercising (once a day), for purposes related to medical needs or caring for the vulnerable, or travelling to and from work (if absolutely necessary). Such restrictions were also enforced at similar times across Wales (Drakeford, 2020), Scotland (Coronavirus (Covid-19) update: First Minister’s speech - 24 March 2020 - GOV.SCOT, 2020)and Northern Ireland (Executive approves new powers to protect the public - The Executive Office NI - 28 March 2020, 2020). On the 23rd of June these measures were eased in England (PM Announces Easing of Lockdown Restrictions: 23 June 2020 - GOV.UK, 2020). Many businesses were able to re-open and members of two separate households were permitted to meet with strict social distancing measures still in place. Various social activities, from meeting large groups of friends inside of the home to larger scale events, are unlikely to be feasible in the usual manner for the foreseeable future. Due to the uncertainty regarding how long these restrictions may be in place for, people may find more security and comfort in nostalgia, through revisiting past lives and reminiscing about times before social distancing measures and restrictions existed. We furthermore suggest that by engaging with music-evoked nostalgia as means to regulate emotions, levels of wellbeing may be improved.

The term *nostalgia* can be defined by looking at its root words: ‘nostos’ and ‘algos,’ constructing its ancient meaning as the suffering caused by the yearning to return to one’s provenance (Wildschut et al., 2006, p. 3). Nostalgia was once employed as an equivalent to the German term, *Heimweh*, described as a state of moral pain resulting from forced separation from one’s homeland, and seen as a cerebral disease affecting Swiss mercenaries or a psychiatric condition (Fuentenebro de Diego and Valiente Ots, 2014). There has been now a shift from the way nostalgia was viewed in the past, as some sort of disease of the mind, to its current consideration as a bittersweet emotion. For the purposes of this study, we will use a more recent andnuanced definition provided by the *Oxford English DictionaryOnline*: ‘Sentimental longing *for* or regretful memory of a period of the past, esp. one in an individual’s own lifetime; (also) sentimental imagining or evocation of a period of the past’ (OED Online, 2020). This definition encompasses the complex affective, narrative and subjective content of the emotion in a way that is highly relevant to the research presented here. Further intricacies continue to be revealed surrounding the nature of nostalgic affect, potential functionality, and the impact of music-evoked nostalgia on wellbeing.

Several recent studies have captured benefits of music listening in general for wellbeing and emotion regulation with regards to the COVID-19 pandemic. Globally, music has held prominence in facilitating social connectedness and emotion regulation in response to COVID-19 (Granot et al., 2021). Individuals have selected music as means to cope with psychological distress, which has served as important means for improving wellbeing (Mas-Herrero et al., 2020), and cope with periods of social isolation (Krause et al., 2021). For those who have experienced an increased negative mood as a result of the pandemic, music listening has helped them to cope emotionally, and regardless of changes in emotions, music has provided a sense of social connectedness for all (Fink et al., 2021). Similar coping effects of music listening have been reported related to relaxation, raising mood, escapism, and social surrogacy, as well as an increased perception of the general value of musicfor wellbeing during lockdown (Cabedo-Mas et al., 2021). In close relation to our research, a study conducted by Yu-Cheong Yeung (2020) has demonstrated the existence of a link between the COVID-19 pandemic and an increase in listening to music deemed nostalgic on the music streaming service Spotify.

Nostalgia has been said to arise in response to feelings of uncertainty or anxieties (Davis, 1977), or as means to find continuity in times of change (Batcho, 2013). Many studies identify a causal link between loneliness and nostalgia (Routledge et al., 2011; Sedikides et al., 2008; Zhou et al., 2008). Lonelier participants report higher levels of nostalgia, and are more likely to turn to nostalgia to provide social support, counteracting their loneliness (Sedikides and Wildschut, 2016). In addition to this, music is powerful evocator of nostalgia (Garrido, 2016; Juslin et al., 2008; Wildschut et al., 2006). An experience sampling study on everyday music listening habits found that *Nostalgia-Longing* was most frequently experienced when listening to music alone, and occurred most commonly during relaxation (Juslin et al., 2008). Music-evoked nostalgia has been identified as linking to solace and comfort, where memories of significant people or times that brought happiness provide calm and comfort, creating a sense of safety and acceptance (Saarikallio, 2011). This evidence substantiates the potential functionality nostalgia has during the COVID-19 crisis, as our previous levels of social connectedness seem more distant than ever.

### Affective and Narrative Content of Nostalgia

The narratives and memories brought about by nostalgic music-listening fuel the emotional affect, and in turn the function it serves. There is evidence supporting the role of reminiscence and nostalgia in social cohesion and connectedness, reminding individuals of certain people, places, or events (Lonsdale and North, 2011). Kikuchi and Noriuchi (2017), also discuss nostalgia as an emotional state involving predominantly positive emotions and provide neurological evidence describing how memory stimulates reward system regions in the brain. This relates to a component of Juslin’s theoretical model for music induced emotions, *Episodic Memory* (2016). Memories of valued past events evoked by music induces both emotions felt in response to those memories and physiological responses stored from those events (Juslin, 2016). Memories and narratives evoked by nostalgia described by Wildschut et al. (2006) were typically associated with other people and momentous events, that were reflected on in a positive sense regardless of the retrospective emotion; even negative memories were alleviated by a sense of triumph (Wildschut et al., 2006). When this music is self-selected, it is likely that interindividual differences of wellbeing states influence what music is chosen and in turn what memories, narratives and affects are evoked.

Cavanagh et al. (2015) conclude that the degree of valence felt when nostalgia is triggered depends upon levels of attachment insecurity. Those who felt insecure about their relationships with others responded to their felt nostalgia in a more negative way, and those who had secure attachments responded positively. Overall, evidence suggests that the emotional impact of music-evoked nostalgia, may depend on trait and state level individual differences. For those with a higher capacity for emotion-regulation, improvements to mood and wellbeing may be achieved, whereas for ruminators who experience depression, nostalgic absorption may be disadvantageous to their mood and wellbeing (Garrido and Schubert, 2013). This experience is therefore entirely dependent on subjective influencing factors, which determine the extent of positive or negative emotion resulting from nostalgia, and in turn the emotion regulatory function nostalgia serves in improving wellbeing.

### Functions of Music-Evoked Nostalgia in Improving Wellbeing

Wellbeing is difficult to define, and as a result is typically assessed as a combination of facets. For instance, the Short Warwick Edinburgh Wellbeing Scale (SWEMWBS) considers there to be 7 facets; optimism, usefulness, ability to relax, resilience, thinking clearly, closeness to others, and decisiveness (Tennant et al., 2006). In consequence, the aims and outcomes of studies into the functions of nostalgia and music are rarely concerned with wellbeing as a higher order concept, but rather the facets the wellbeing encompasses.

Sedikides and Wildschut (2016) discuss the psychological health benefits and functions of nostalgia in broad categories of sociological, existential and self-related, as well as the potential it has for buffering against psychological threats in any of these categories. Not only was nostalgia seen to increase social connectedness, but it also improved self-esteem, optimism, sense of purpose and meaningfulness in life, as well as alleviating boredom. In addition, those with higher ratings of nostalgia were able to take more responsibility for failure, demonstrated higher fortification against self-threat, and showed reduced levels of stress (Sedikides and Wildschut, 2016). In a study on terror management theory and mortality salience, feelings of nostalgia were found to be used as a self-protection mechanism through reflecting on the past as means to reconstruct world-view, and reinforce meaning when faced by threat (Routledge et al., 2008). Conversely, Verplanken (2012) found that absorption in the past, even if it were positive, may not be beneficial for habitual worriers, as positive memories could turn into negative thoughts and cause distress as a result of failing self-regulation and depressive rumination. Qualities of fortification and resilience are reliant on adaptive coping and emotion regulation strategies, however, in some cases, poor mental and emotional health impede ability to self-regulate.

Music listening can be a fundamental resource for emotion regulation. This is especially the case in relation to moments of social or economic crisis. Pettijohn and Sacco (2009) found that during times of social or economic threat and instability, music that is comforting, or provides an outlet in which fears and uncertainties may be explored is preferred. Listening is commonly used to combat negative mood and escape from problems related to poor wellbeing. Specific emotional goals can be realised through careful music selection, based upon the desires and needs of the individual (Randall et al., 2014). Randall and Rickard (2017) found that emotion regulatory reasons behind personal music listening were present only when participants were in negative moods. As a result, listening is used to realise certain emotional needs, determined by momentary mood and influenced by general emotional health. Therefore, participants who are initially in a negative mood may improve it using music, or deliberately maintain their negative mood using music, as a result of emotional instability and poor mental health.

Subjective coping strategies through listening can also comprise of music that evokes nostalgia (Batcho, 2013; Garrido, 2016; Garrido et al., 2016). Garrido (2016) states the resulting positive or negative emotions may depend on an individual’s nostalgic habits and how they view the past. They conclude that although previous studies recognise that people who feel depressed are attracted to nostalgia and can often respond positively to engagement with it, those with clinical depression may also find patterns of negative thinking intensify, and maladaptive coping strategies based upon denial of the present reality could arise. Several studies conducted by Routledge et al. (2011) demonstrated that music-evoked nostalgia was associated with feelings of being loved, and reading nostalgic lyrics induced a sense of meaning and purpose in life, and increased social connectedness. Similar findings were evident in a study by Schäfer and Eerola (2020), whereby music that induced nostalgia was identified as being a powerful mechanism for social surrogacy and combatting loneliness.

The *Emotion Regulation Strategiesfor Artistic Creative Activities (ERS-ACA)* model, developed by Fancourt et al. (2019), might be well suited to describing the adaptive strategies and positive emotive outcomes associated with nostalgic absorption. It contains 3 factors representing different emotion regulation strategies: *Avoidance*, by means of distraction and escapism from difficult and problematic experiences; *Approach*, by means of actively confronting and dealing with experiences in the present; and *Self-Development* by means of improving and reaffirming a sense of self. The scale which this model is derived fromis designed to assess different strategies in how individuals regulate their emotions, rather than observing adaptive or maladaptive functioning, which Fancourt et al. (2019) argue have unclear divisions and can be changeable. Instead, adopting emotion regulation strategies are thought to be generally linked to more positive mental health and wellbeing, regardless of whether a strategy might consist of avoidance in order to cope with stress or anxiety (Kashdan et al., 2014). Overall, nostalgia has been shown to be a meaningful resource, which can be harnessed as a tool for reflection and development. To quote Batcho (2013), ‘Longing for the past is particularly relevant as people struggle for a sense of continuity in a rapidly shifting landscape or their personal and social lives’ (p. 9). Where the present and future are rife with uncertainty, the reassurance of the past could hold certainty that may sustain a sense of meaning and purpose in life through these unprecedented times.

### Aims

For these reasons, we predict that nostalgic music listening is prevalent through the COVID-19 crisis, and that it can form an emotion regulation strategy leading to increased wellbeing. Accordingly, this study aims to investigate individual experience of music-evoked nostalgia in relation to wellbeing during the COVID-19 crisis in the United Kingdom, through exploring emotional and narrative content, functions, and overall effects of music-evoked nostalgia during the enforcement of lockdown restrictions. To investigate this, two main research questions will be considered.

*RQ 1* How frequently have participants engaged with nostalgic music during the initial lockdown, and how can the narrative and affective content of their listening experiences be captured?

*RQ 2* Is the affective and narrative content of music-induced nostalgia associated with emotion regulation strategies, and can the latter lead to higher levels of wellbeing?

## Materials and Methods

### Participants

Participants were recruited through University of York department mailing lists, word of mouth, and through social media. In total 570 recruits completed an online questionnaire. One requirement for participants was that they had to have lived in the United Kingdom for at least the majority of the first lockdown. Although different areas of the United Kingdom enforced a lockdown at different times and to varying levels, the dates specified were between the 23rd March and 23rd June, which corresponded to the initial introduction of the lockdown and ‘stay at home’ rhetoric across England (Prime Minister’s Statement on Coronavirus (COVID-19): 23 March 2020 - GOV.UK, 2020), through to the general easing of the measures for most (PM Announces Easing of Lockdown Restrictions: 23 June 2020 - GOV.UK, 2020). The participants were asked which country they resided in for the duration of the stated period by selecting from a set list of United Kingdom regions (87% resided in England, 6.5% in Wales, 6% in Scotland, and 0.5% in Northern Ireland). The majority of participants were therefore subjected to English lockdown parameters. 33.5% of participants identified as male, 65.6% female, 0.4% as non-binary and 0.5% preferred not to say. Ages ranged from 18 to 84 years, with 7 participants choosing not to share their age (*M* = 44.20, *SD* = 15.801).

Other demographic information included musicianship, work status, and living circumstances. Musicianship was ascertained on a scale from non-musician to professional, with levels in-between that aligned with ABRSM grade standards (43.3% non-musicians, 7% beginner, 13.5% amateur, 10.2% intermediate, 9.1% higher level, 11.4% university student or semi-professional, 5.4% professional). Work status was selected from 7 categories (11.2% student, 46.8% employed, 3.2% unemployed, 15.8% furloughed or temporarily out of work, 12.6% retired, 3.3% disabled, and 7% other, including carers, volunteers, and self-employed or freelance).

Finally, living circumstances were assessed in 3 categories, whether alone, with family, or with friends or acquaintances, and had the option of self-describing to further explain their circumstances (13.7% living alone, 78.9% with family or close knit household involving those living with family and/or partners, and 7.4% in households that were considered to be less close knit, consisting of friends, shared university accommodation, and 7.4% households with people described as ‘lodgers,’ ‘tenants,’ ‘roommates,’ ‘housemates,’ or ‘flatmates’). It is also worth noting that many participants and stated they had moved at the start of lockdown or during, in order to avoid being alone.

### Procedure and Measures

The online questionnaire was created using Qualtrics and had three sections: (i) general wellbeing and life impairment through the period of lockdown, (ii) general functions of nostalgic music and a self-selected nostalgic music listening task, and (iii) personality and demographics. Section (i) was used to address RQ2, which was presented to participants first in order not toinfluencethese results by the listening task or completion of demographic questions. Section (ii) addressed both research questions, in both the experience nostalgic music listening for RQ1 and the function it served for RQ2. Section (iii) was used to describe the sample. A pilot study was conducted with 10 participants, to ensure the accessibility effectiveness of the questionnaire.

Section (i) assessed general levels of wellbeing using the *Short Warwick-Edinburgh Mental Wellbeing Scale* (Tennant et al., 2006), and the extent that the COVID-19 lockdown had impaired aspects of work and social functioning using a version of the *Work and Social Adjustment Scale (WSAS)* (Mundt et al., 2002), which was adapted for specific use in this questionnaire. This version is included in Supplementary Material 1, and our adaptation included 3 additional items. Typically, this scale is designed to determine the extent that a problem an individual is facing impairs their ability to carry out various activities. The problem in this situation was the same for all participants, as they were asked to think back to how they felt during the strictest period of the lockdown, between the 23rd March and 23rd June.

For section (ii) general functionality of nostalgic music was assessed using the *Emotion Regulation Strategies for Artistic Creative Activities*scale (*ERS-ACA*) (Fancourt et al., 2019), which was presented specifically in response to engaging with nostalgic music listening, in order to determine participants’ use of music-evoked nostalgia as a strategy. The full list of the emotion regulation (*ERS-ACA)* items is presented in the factor loading table Supplementary Material 2. Participants were provided the following background information explaining the concept of nostalgia and nostalgic music, prior to rating emotion regulation statements and engaging with the subsequent music listening task:

*The emotion of nostalgia is defined by The New Oxford Dictionary of English (1998) as ‘a sentimental longing or wistful affection for the past’ (p. 1266). Nostalgia can make you feel both good and bad, whether you mourn the past or look backward to remind you of the happiness you have felt in your life. ‘The nostalgizer, then, is presumed to feel negatively for a bygone way of life, for the passing of treasured moments, and for the current absence of persons significant to them. At the same time, the nostalgizer feels positively for having had the opportunity to share defining life events with those significant others.’* (Sedikides and Wildschut, 2016) *Nostalgic music is defined as music which gives you feelings of nostalgia, that is, feelings of sentimental longing for the past. The next series of questions relate to your experience of nostalgic music. This could be music from specific times of your life, associated with certain people, or music you listened to when you were younger. It may be bittersweet, positive or negative, or a mixture of the two.*

After rating their agreement with emotion regulation (*ERS-ACA)* statements, they were then asked the frequency in which they had listened to nostalgic music over the period of lockdown. If they had, they were then asked to select a piece of music that induced nostalgia that they had listened to during the lockdown. All music selections given by participants are provided in Supplementary Material 3. Following this selection, they were asked to provide a summary of their associated memories with the piece of music, and then listen to the piece of music. After listening, they were asked to describe their immediate emotional response to the music in one word, then to rate a series of statements on their experience of this selected music. These statements were taken from Garrido’s (2016) questionnaire of positive or negative nostalgic effects, *Experienced Effect of Nostalgia* (*EEN*), capturing quantitative measures of the momentary experience of music-evoked nostalgia on a 7-point scale (strongly disagree to strongly agree). The statements are given in response to the question, ‘How did listening to this music make you feel?,’ and are as follows:

It made me happy to think about happy times in the past. It made me sad because those happy times I had in the past are gone. It made me appreciate where my life is now because I have come so far. It made me sad remembering difficult times in the past. It reminded me of people that I used to see more often, and I enjoyed thinking about them and feeling more connected to them. The experience was bittersweet, somewhat happy and somewhat sad.

Section (iii) included the *Ten Item Personality Inventory* (*TIPI*) (Gosling et al., 2003) and demographic questions.

### Data Analysis

Statistical analyses were conducted using SPSS (version 26.0), JASP (version 0.13.1) for Confirmatory Factor Analysis, and R (version 4.0.2) to extract factor scores using the lavPredict function and Lavaan syntax resulting from the JASP CFA. Graphical figures were produced using OriginPro (version 9.7).

## Results

### Research Question 1: Frequency of Nostalgic Listening and Capturing the Experience of Music-Evoked Nostalgia

#### How Frequently Have Participants Been Listening to Nostalgic Music During Lockdown?

First, frequency of listening during the period of lockdown was determined, in order to gain some sense of the prevalence of nostalgic listening during the COVID-19 crisis. The question of frequency of listening was presented to participants in the form of a 6-point Likert scale, ranging from ‘Never’ to ‘At least once a day’. A large majority of participants claimed to have listened to nostalgic music at least once a week for the duration of lockdown, in comparison to just over 1% stating that they never have (see Table 1). Those who had expressed they never listened to nostalgic music through the lockdown skipped the following listening task.

**TABLE 1:** How often have you found yourself listening to nostalgic music, either intentionally or coincidentally, over the last 3 months?

	Frequency	Percent	Cumulative percent
At least once a day	117	20.5	20.5
At least once a week	271	47.5	68.1
At least once a fortnight	85	14.9	83.0
At least once a month	60	10.5	93.5
Less than once a month	28	4.9	98.4
Never	9	1.6	100.0
Total	570	100.0	

#### What Is the Affective and Narrative Content of Nostalgic Experiences?

Participants then reported a piece of self-selected music that they had listened to during lockdown that induced nostalgia. While the survey requested that participants identify self-selected songs, 15.9% of participants referred to music in other formats, including albums, artists, genres, radio stations, and recorded concert sets. Prior to listening, they were asked to describe the possible reasoning behind why it might have made them feel nostalgic, and the content or memories associated with these feelings. Following the listening task, they were required to provide one word that summarised their immediate emotional response.

##### Thematic analysis of nostalgic reasoning

Thematic analysis of participants’ nostalgic reasoning for their self-selected music generated 25 categories. These categories were organised into ten higher-order categories which summarised the content or narratives of participants’ experienced nostalgia. These categories are described below, along with the percentage of participants assigned to each category.

###### A general period of life

A total of 54.5% of participants’ responses referred to a general period of their past life. Common themes were childhood surrounded by family, or university years. One person wrote ‘Reminds me of my uni days, gigging with friends and good times.’

###### Memory of an event or situation

A total of 18% of participants recalled a specific event or situation, which they associated with their selected pieces of music. Some reflected on what was happening in their life at the time of the event, for instance one participant wrote ‘Reminds me of where I was in my life when I saw the band play live.’ Others focused on the memory itself and its meaning to them, for example ‘First dance at my wedding.’

###### Person or people

This category incorporated those who mentioned anyone other than themselves, which was true for 34.4% of participants. Popular themes included family, friends, partners and ex-partners, communities or groups of people. People recalled memories of people who they feel close to, or who they have not seen in years. For example, one wrote ‘Remind me of my father,’ and another ‘Reminds me of a friend who gave me the track who I don’t see much of anymore.’

###### Place or travel

A total of 11.8% of participants chose to reflect on their childhood or university home, a holiday or time spent living abroad, or a memorable journey. From ‘Driving around the north east of Iceland with my Mum in 2009, we were both so happy’ to ‘It reminds me of when I was living in Berlin and myself and groups of friends would go to watch East Berlin’s football team 1. FC Union Berlin.’

###### Perspective

A total of 13.9% of participants were assigned to this category, as they seemed to reflect comparatively on their lives overall or the world around them. Many reflected on times that felt simpler, or times when they felt they had less responsibility. Others discussed the world around them in general. As one person wrote, ‘I’d say it gives me a strong sense of spatial nostalgia, as opposed to something that personally relates to me. It helps me identify with the past and older, and structurally simpler times.’

###### Performance

A total of 8.4% of participants selected music that they have enjoyable memories of performing. Some performances were musical, for instance, ‘I played this piece of music on the clarinet at a memorial party of my parents several years ago,’ and other pieces of music were performed as a DJ or through dance.

###### Music liking

A total of 23.9% of participants selected music that they have always enjoyed or have listened to for some time. Many linked this to their identity and developing tastes, for example ‘It’s from 2016, when I had finally found my own favourite music and wasn’t relying so heavily on the charts and this was the summer I finished secondary school and started college (I felt like an adult).’

###### Triumph

A total of 1.8% of participants referred to feeling of triumph as a result of emerging from a difficult time. One participant wrote ‘It reminds me of the freedom I felt when my husband finally decided to leave me. I’d been unhappy and trapped for a long time.’

###### Trauma

A total of 8.4% of participants provided details of trauma that was linked to their nostalgic song selections. Some of these detailed funerals they attended or bereavements, for instance one was reminded of ‘the baby daughter I held in my arms who died a few weeks later.’ Others recalled times where they suffered as a result of their mental health. One participant’s self-selected nostalgic song reminded them of ‘self-harm and dissociating.’

###### Pre-COVID longing

A total of 2.5% of participants were assigned to this category, which were fewer than expected, however, reasoning was categorised here only if they eluded to lockdown, or something not currently possible. One wrote ‘Remember singing it and being sad singing with others is not possible at the moment.’ Another stated they were nostalgic ‘for a time when we could go to festivals, dance, live without fear of getting ill or making someone ill.’ It is likely that many more could have fit within this category, however, whether their reasoning was related to lockdown or not was not always clear enough.

##### Content analysis of emotional responses

Content analysis of participants’ one-word emotional responses to their self-selected music categorised each into one of four valencegroups: (i) positive valence, (ii) negative valence, (iii) mixed or complex valence, (iv) neutral valence. For both (ii) negative and (i) positive groups, participants’ emotions were easily discernible, and largely consisted of ‘sad’ or ‘happy.’ For (iii) mixed or complex valence, emotional responses assigned here were either bittersweet or simply unclear, for instance; ‘wistful’ or ‘sentimental.’ The final category of (iv) neutral valence was included to account for those who indicated little or no emotional response, as some stated they felt ‘OK’ or ‘unchanged.’ Table 2 presents the distribution of frequencies for each coded valence group. As can be seen, a large majority of emotional responses showed positive valence.

**TABLE 2:** Frequency table for coded valence response groups to nostalgic music.

	Frequency	Percent
Never listen to nostalgic music	9	1.6
Mixed or complex emotions	107	18.8
Negative emotions	65	11.4
Neutral emotions	15	2.6
Positive emotions	374	65.6
Total	570	100.0

Following the categorisation of qualitative responses presented here, we tested for associations between the coded valence groups and coded nostalgic reasoning variables, and the quantitative variables of nostalgic effects (*EEN)* (Garrido, 2016).

#### Associations Between Nostalgic Effects (*EEN)*, and Coded Valence Groups and Nostalgic Reasoning

A confirmatory factor analysis was conducted for all 6 nostalgic effect (*EEN*)items (Garrido, 2016), to test whether the original model of two factors was a reasonable fit for the observed data. The results of this demonstrated poor fit across all indices, *X*^2^ (9, *N* = 561) = 132.493, *CFI* = 0.793, *TLI* = 0.656, *RMSEA* = 0.156, *SRMR* = 0.097, and therefore factors could not be satisfactorily extracted. Exploratory factor analysis was then conducted to try and ascertain a model with better fit. The results were not conclusive for both methods of parallel analysis and Eigenvalues greater than one, and the resulting factors did not seem consistent with the theoretical background. Both the variables of *Appreciation*, which describes appreciating ‘where my life is now because I have come so far,’ and *Bittersweet*, which describes the experience as being ‘somewhat positive, somewhat negative,’ seem to be too semantically different from the other positive or negative statements. Table 3 shows the Pearson correlation matrix for all nostalgic effect variables. As this matrix shows, the size of correlations between variables were not high enough for a factor analysis approach to be applicable to the dataset. Subsequently, each of the nostalgic effect variables were analysed separately.

**TABLE 3:** Pearson correlation matrix for nostalgic effects (*EEN)* (Garrido, 2016).

Variable		*Happy past times*	*Sad past times*	*Appreciation*	*Sad difficult times*	*Reminder of people*	*Bittersweet*
*Happy*							
*Past times*	*r*	–					
*Sad past times*	*r*	0.01	–				
*Appreciation*	*r*	0.189***	−0.187***	–			
*Sad difficult times*	*r*	−0.175***	0.332***	0.108*	–		
*Reminder of people*	*r*	0.405***	0.225***	0.158***	0.117**	–	
*Bittersweet*	*r*	–0.029	0.54***	0.029	0.492***	0.234***	–

*p <0.05,**p < 0.01,***p < 0.001, *n* = 561.

##### Nostalgic effects (EEN) and coded valence groups

**FIGURE 1:**
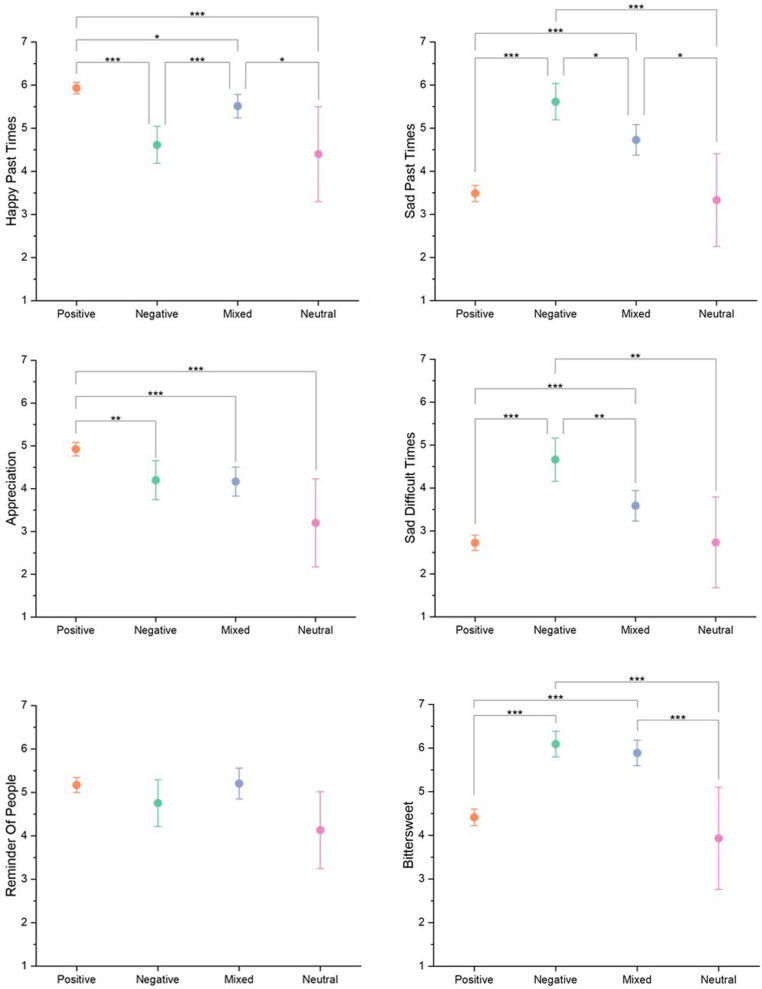
Tukey HSD post hoc paired comparisons for nostalgic effects (EEN items) and coded valence groups with 95% Confidence Interval error bars.*p <0.05, **p <0.01, ***p <0.001, *n* = 561.

Table 4 presents ANOVA results testing whether the coded valence groups were associated with nostalgic effects (EEN). As shown, for almost all nostalgic effects, there were significant differences in the means between coded valence groups, with exception of Reminder of People, which showed a non-significant trend. Post hoc paired comparisons using Tukey HSD test were conducted to explore associations between coded valence groups (factor variable) and nostalgic effects (outcome variables). These are represented as error bar graphs in Figure 1 (see Supplementary Material 4 for full results).

**TABLE 4:** Analysis of Variance table for nostalgic effects (*EEN)* and coded valence groups.

Dependent	Source	d*f*	*F*	*p*	η*2*
*Happy past times*	Between valence groups	3	21.456	< 0.001***	0.104
	Within groups	557			
*Sad past times*	Between valence groups	3	33.106	<0.001***	0.151
	Within groups	557			
*Appreciation*	Between valence groups	3	12.066	<0.001***	0.061
	Within groups	557			
*Sad difficult times*	Between valence groups	3	24.294	<0.001***	0.116
	Within groups	557			
*Reminder of people*	Between valence groups	3	2.612	0.051^†^	0.014
	Within groups	557			
*Bittersweet*	Between valence groups	3	32.476	<0.001***	0.149
	Within groups	557			
	Total	560			

***p < 0.001, ^†^non-significant trend at *p* < 0.10, *n* = 561.

For the *Happy Past Times* statement, significant differences between means were found amongst all post hoc paired comparisons of the positive, negative, and mixed groups. Mean ratings of this statement for both positive and mixed groups were significantly higher than that of the neutral and negative groups, although the mean rating for the positive group was only marginally higher than the mixed. Regarding the *Appreciation* statement, the mean rating of the positive valence group rated this nostalgic effect the highest, which was significant in comparison to the other valence groups. No significant difference was observed between paired comparisons of other groups. Participants with emotional responses that were coded into positive or mixed valence groups both rated the nostalgic effect *Reminder of People* comparatively highly, although no significant difference found between any of the paired comparisons.

The mean scores for the negative valence group’s ratings of the nostalgic effects of *Sad Past Times* and *Sad Difficult Times* were found to be significantly higher than all other groups’ means. The mixed valence groups’ mean ratings for both were also significantly higher than positive and neutral. Interestingly, the *Bittersweet* nostalgic effect was agreed with comparatively for participants in both negative and mixed valence groups with no significant difference identified between their ratings. Participants in the positive and neutral valence groups agreed with the Bittersweet nostalgic effect statement significantly less.

##### Nostalgic effects (EEN) and coded nostalgic reasoning

Relationships between nostalgic effects and all coded categories of reasoning were explored through multiple linear regression, and the coefficient estimates are presented in Table 5.

**TABLE 5:** Linear regression coefficient estimates for predicting nostalgic effects (*EEN* items) (outcome variables) with coded reasoning categories^1^ (predictor variables).

	*Happy past times*	*Sad past times*	*Appreciation*	*Sad difficult times*	*Reminder of people*	*Bittersweet*
	β	β	β	β	β	β
*Period of life*	0.105*	–0.040	0.035	–0.059	0.042	0.012
*Event, situation memory*	0.099*	0.034	0.001	–0.039	0.079†	0.009
*Person or people*	0.107*	0.219***	–0.034	0.022	0.219***	0.132**
*Place or travel*	0.108*	0.038	–0.006	–0.049	–0.006	0.060
*Perspective*	0.046	0.048	0.099*	0.058	0.039	0.118**
*Performance*	0.017	0.090*	0.018	0.010	0.085*	0.072
*Music liking*	0.002	< 0.001	0.018	0.027	–0.039	0.015
*Triumph*	–0.062	–0.041	0.085*	0.020	0.046	–0.016
*Trauma*	−0.100*	0.064	0.039	0.194***	0.084*	0.162***
*Pre-COVID longing*	0.079†	–0.003	0.040	–0.019	0.089*	0.072
*F*(10,550) =	3.291***	4.472***	1.342	3.176**	5.181***	3.770***
Adjusted *R*^2^	0.039	0.058	0.006	0.037	0.069	0.047

*p <0.05, **p <0.01,***p <0.001. †non-significant trend at *p* <0.10. ^1^Recoded as dummy variables, *n* = 561.

The significant relationships here all had small effects and many of them are as expected. For example, when participants provided narratives related to happy times from participants’ pasts, including general period of life, events or situations, places or travel, and being reminded of people, they were significantly more likely to agree with the *Happy Past Times* nostalgic effect statement. In turn, participants who reported trauma were significantly less likely to agree with the *Happy Past Times* nostalgic effect, and more likely to agree with *Sad Difficult Times.* Participants’ who gave nostalgic reasoning that related *Person or People*, and *Performance* were significantly more likely to agree with the nostalgic effect (*EEN*) of *Sad Past Times*. Furthermore, participants who provided nostalgic reasoning which fulfilled categories of *Perspective* and *Triumph* were significantly more likely to agree with the nostalgic effect statement of *Appreciation* Participants who agreed with the nostalgic effect statement, *Reminder of People* were significantly more likely to provide nostalgic reasoning which related to *Person or People, Performance, Trauma* and *Pre-COVID-Longing*. Finally, participants who agreed with that they related to the *Bittersweet* nostalgic effect were significantly more likely to provide reasoning which related to the categories of *Person or People*, *Perspective*, and *Trauma.*

### Research Question 2: Functions of Nostalgic Music as Emotion Regulation Strategies and Their Associations With Wellbeing

Having explored the affective and narrative content induced by nostalgic music, the second research question aimed to address whether this content is related to emotion regulation strategies. This was investigated using the emotion regulation (*ERS-ACA)* scale (Fancourt et al., 2019).

#### Emotion Regulation (*ERS-ACA)* Confirmatory Factor Analysis

**FIGURE 2:**
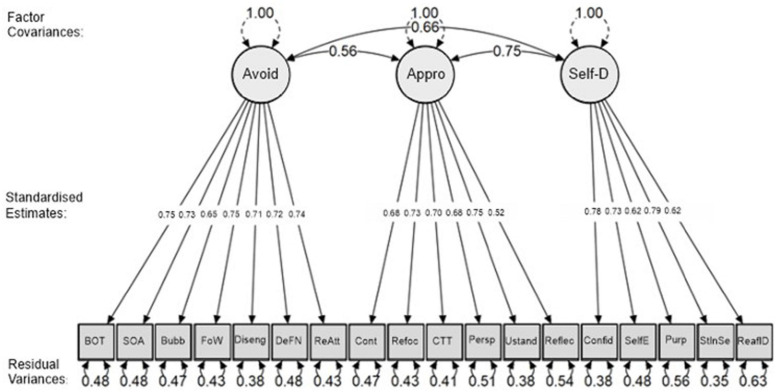
Confirmatory factor analysis model of emotion regulation strategies.

Confirmatory factor analysis of the ratings of theemotion regulation statements provided comparable results to that of the original study (Fancourt et al., 2019). All fit indices supported the model as being significantly applicable for the observed data, *X*^2^ (132, *N* = 570) = 352.372, *CFI* = 0.952, *TLI* = 0.944, *RMSEA* = 0.054, *SRMR* = 0.050. All standardised estimates loaded between 0.5 and 0.8, meaning no items required removal. The model is presented below as Figure 2 (Supplementary Material 2 presents factor loadings of all emotion regulation (*ERS-ACA)* items). All three emotion regulation factors – *Avoidance, Approach and Self-Development* - were positively correlated with each other, indicating that the likelihood of engaging with one particular emotional regulation strategy increases with the likelihood of the two other emotion regulation strategy.

#### Emotion Regulation (*ERS-ACA)*Association With Nostalgic Effects (*EEN*)

For the next step in addressing the second research question, a multiple linear regression was conducted using the nostalgic effects (*EEN)* as outcome variables, and emotion regulation factor scores (*ERS-ACA)* as predictor variables, in order to see if specific items related more to certain emotion regulation strategies than others. Table 6 shows the coefficient estimates from this analysis.

**TABLE 6:** Linear regression coefficient estimates for predicting nostalgic effects *EEN* items (outcome variables) with emotion regulation (*ERS-ACA)* factors (predictor variables).

	*Happy past times*	*Sad past times*	*Appreciation*	*Sad difficult times*	*Reminder of people*	*Bittersweet*
	β	β	β	β	β	β
*Avoidance*	0.247***	−0.158*	0.104†	−0.186**	0.037	−0.190**
*Approach*	0.079	0.111	0.297***	0.237**	0.115	0.105
*Self-development*	0.018	0.008	–0.005	–0.026	0.167*	0.139
*F*(10,550) =	20.845***	2.887*	28.874***	6.433***	18.085***	5.286**
Adjusted *R*^2^	0.096	0.010	0.130	0.028	0.084	0.022

*p < 0.05, **p < 0.01,***p < 0.001, † non-significant trend at *p* < 0.10*n* = 561.

This analysis showed that the experience of nostalgic music for those who use it as an *Avoidance* emotion regulation strategy involves predominantly thinking about happy times from the past. There was a negative association between *Avoidance* and all negative nostalgic effects, seemingly showing that for this strategy participants prefer to focus on the positive aspects of their life in order to avoid the present. *Approach* presented statistically significant positive associations with both *Appreciation* and *Sad Difficult Times*. Participants who adopted the *Self-Development* strategy were only significantly likely to experience the *Reminder of People* nostalgic effect.

#### Emotion Regulation (*ERS-ACA)*Association With Coded Reasoning and Valence Categories

We then tested if and how the coded reasoning categories and the coded emotion groups from 3.1.2, were associated with emotion regulation strategies. Full linear regression coefficient estimates for reasoning category dummy variables predicting emotion regulation factor scores are provided in Supplementary Material 5. Means and standard deviations for emotion groups of each emotion regulation factor are presented in Table 7.

**TABLE 7:** General linear model univariate tests for coded valence categories (predictor variable) and emotion regulation (*ERS-ACA)* factors (outcome variables).

Variable		Mean	*SD*	*n*	d*f*_1_	d*f*_2_	*F*	*p*	Partial η*2*
*Avoidance*					3	557	10.553	<0.001	0.054
	Positive	0.117	0.703231	374					
	Negative	–0.128	0.811588	65					
	Mixed	–0.214	0.695407	107					
	Neutral	–0.583	0.707007	15					
*Approach*					3	557	5.773	0.001	0.030
	Positive	0.059	0.584664	374					
	Negative	–0.033	0.641602	65					
	Mixed	–0.062	0.563217	107					
	Neutral	–0.529	0.488616	15					
*Self-development*					3	557	6.878	< 0.001	0.036
	Positive	0.08160	0.704497	374					
	Negative	–0.15115	0.782687	65					
	Mixed	–0.06549	0.615078	107					
	Neutral	–0.60966	0.582824	15					

*n = 561.*

For the coded reasoning categories, participants who indicated that theytreated nostalgic music listening as an *Approach* strategy were significantly more likely to provide nostalgic reasoning that related to the *Perspective* category (β = 0.097, *t* = 2.232, *p* =0.026). Participants who indicated they used nostalgic music to regulate their emotions by adopting the *Self-Development* strategy were significantly more likely to provide nostalgic reasoning that related to the *Period of Life* category (β = 0.096, *t* = 2.064, *p* = 0.039). The only other relationships were non-significant trends, whereby participants using the *Approach* may have been more likely to provide nostalgic reasoning relating to the *Person or People* category, and participants who indicated reliance on the *Self-Development* strategy may have been more likely to provide reasoning that related to the coded reasoning categories of *Event, Situation or Memory* or *Perspective*.

**FIGURE 3:**
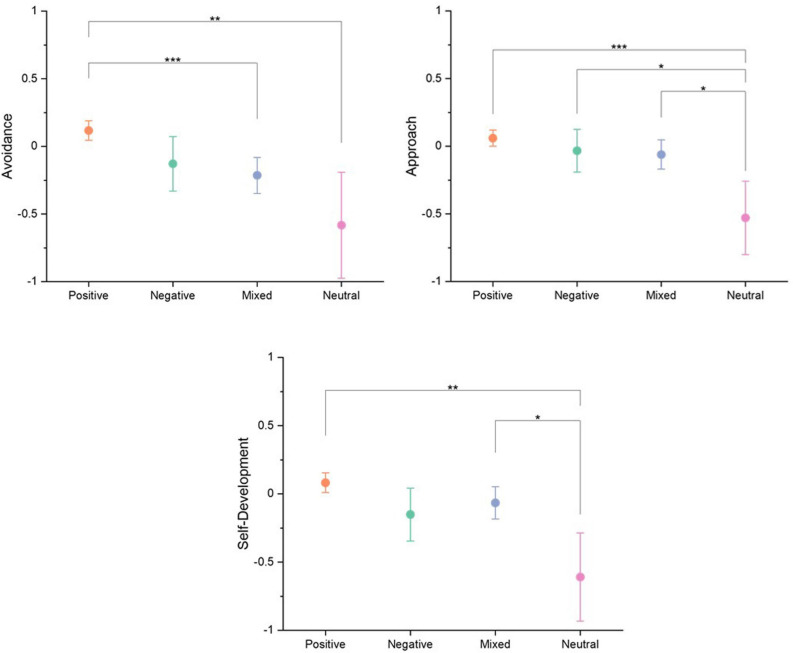
Tukey HSD post hoc paired comparisons for emotion regulation (ERS-ACA) factors and coded valence groups with 95% Confidence Interval error bars. *p <0.05, **p <0.01,***p <0.001, *n* = 561.

For the coded emotion groups, post hoc Tukey HSD comparisons were conducted for each emotion regulation factor, which are graphically presented in Figure 3 (see Supplementary Material 4 for full results). These comparisons showed that participants whose emotional responses were coded into the Positive valence group were more likely to have indicated their nostalgic music listening was used as an *Avoidance* strategy than the Mixed and Neutral groups. Participants who indicated little to no emotional response, and were coded into the Neutral valence group, were significantly less likely to have adopted an *Approach*strategy in comparison to the other valence groups. Lastly, participants who indicated their treatment of nostalgic music listening relied on the *Self-Development* strategy were significantly more likely to have provided an emotional response that was codedintothe Positive valence group and Mixed group than the Neutral valence group.

#### Relationship Between Emotion Regulation Functions of Nostalgia and Levels of Wellbeing

The final part of the second research question aimed to test the effect that the different emotion regulation strategies employing nostalgic music had on levels of wellbeing.

Table 8 presents the results from a stepwise linear regression approach, using the emotion regulation (*ERS-ACA)* factor scores as predictor variables, wellbeing (*SWEMWBS)* as an outcome variable. Since a participant’s individual level of impairment of the lockdown was unlikely have been modified by listening to nostalgic music and very likely to influence their overall wellbeing, we included responses to the impairment (*WSAS*) scale as a covariate to the model to control for any underlying interindividual differences in impairment.

**TABLE 8:** Stepwise linear regression coefficient estimates for predicting wellbeing (*SWEMWBS)* through emotion regulation strategies (*ERS-ACA* factor score variables) and impairment (*WSAS)^1^*(as covariate).

Model		Adjusted *R*^2^	β	*T*
1	(Intercept)	0.000		136.056***
2	(Intercept)	0.268		84.172***
	Impairment (WSAS) Total Score		–0.518	−14.437***
3	(Intercept)	0.278		84.646***
	Impairment (WSAS) Total Score		–0.526	−14.701***
	CFA Approach Score		0.100	2.782**

**p < 0.01, ***p < 0.001. The following predictor variables were considered but not included in the final models: CFA Avoidance Score, CFA Self-Development Score. ^1^Values based on sum score, *n* = 570.

This final resulting regression model shows that the impairment (*WSAS)* variable accounted for over a quarter of the explained variance in wellbeing (*SWEMWBS)*, whereby participants with lower levels of life impairment resulting from the COVID-19 lockdown restrictions generally had higher scores of wellbeing. The only other significant predictor of wellbeing was the emotion regulation (*ERS-ACA) Approach* factor, which had a highly significant positive association with wellbeing and contributed an additional 1% of the overall explained variance. In general, this analysis shows that by listening to nostalgic music with an *Approach* strategy in mind, wellbeing is highly likely to be improved, even if the effect of this association is small.

## Discussion

The circumstances of this study were remarkable, in that a crisis such as this has never been experienced before, and therefore no specific hypotheses were made and instead broader research questions were proposed. The first research question addressed how frequently participants had engaged with nostalgic music, and what the narrative and affective content of music-evoked nostalgia might be in relation to the COVID-19 crisis. The second questioned whether the use of nostalgic music as emotion regulation strategies might have comprised of different affects and narratives, and whether the use of these strategies resulted in higher levels of wellbeing.

### Research Question 1: Frequency of Listening, and Narrative and Affective Content

Overall, it is indisputable that a large majority of people have engaged with nostalgic music to some extent over the course of lockdown. As this study positioned its questions specifically within the context of the lockdown, it was not clear whether this was a greater or lesser amount than they otherwise would have. However, from the investigations on the affective and narrative content of nostalgia, it seems that people may have had more to be nostalgic about during the current COVID-19 crisis. We assume that in normal circumstances, people may not have selected pieces that reminded them of people they would usually see in daily life, or selected pieces that they missed performing, however, this is purely conjecture.

Firstly, in relation to the coded emotional content, positive emotional responses were identified as most prevalent when engaging with music-evoked nostalgia during the listening task. Presumably this is because the music listened to was self-selected by the participants, and therefore was likely to arouse positive emotions due to the power of choice. Even so, many participants experienced negative or mixed emotions. This could be due to the distance felt in relation to the things they felt nostalgic for, due to the implementation of social distancing measures, or due to the idea of rumination and depressive tendencies discussed by Garrido and Schubert (2015), whereby those who exhibit higher scores in rumination may have been more drawn to self-selected music that induces negative emotion. This can only be speculation here, as the idea of rumination was not observed in this study.

With regards to the nostalgic effect (*EEN*), *Reminder of People*, no significant difference was observed between the means for any of the coded valence groups, however, there was a non-significant trend of an effect for the overall ANOVA model. This suggests that during lockdown, reminders of people resulting from music-evoked nostalgia may be independent from the degree of valence induced by the music. A possible interpretation of this finding is that reminders of people through music are prevalent due to the separation caused by lockdown, as was found in several recent studies of the role of music during the pandemic for social surrogacy (Mas-Herrero et al., 2020; Fink et al., 2021; Granot et al., 2021). However, any specific emotion evoked by music-evoked nostalgia did not arise as a result of being reminded of people they may have been separated from, but from other variables such as appreciating the past, or acknowledgment of happy past times or sad difficult times.

Feelings such as bittersweet, sentimentality or yearning were all categorised as Mixed valence. The *Bittersweet* nostalgic effect was rated similarly highly for both Negative and Mixed valence categories. Although this is supported by the fact that Garrido et al. (2016) incorporates this *Bittersweet* variable into a factor of Negative Effect, this result seems to contradict Sedikides and Wildschut (2016), and Wildschut et al. (2006) research outlined earlier regarding positive effect outweighing the negative in the context of bittersweet nostalgia. This suggests that for bittersweet nostalgic effect, the positive does not always outweigh the negative, rather, negative valence seems to arise just as much as mixed. However, there does seem to be evidence to suggest that regardless of the momentary nostalgia-induced valence, participants general nostalgic experience suggests notions of optimism and perseverance (Sedikides and Wildschut, 2016). Although negative states may arise through nostalgic narratives, they are filtered through a presently redemptive mindset; past hardships are in the past and the person is able to maintain a positive perspective on them (Davis, 1977).

With regards to the narrative content, some thought-provoking associations were identified. For the *Sad Past Times* nostalgic effect, there was a highly significant positive association with the categories of *Person or People*, and *Performance*. This could be interpreted as being relevant to the current crisis, due to both variables being things affected by social distancing measures and safety restrictions, whereby social in-person interactions in the United Kingdom have been limited, and in turn, performing for musicians in their usual settings (such as in-person orchestra and choir rehearsals) has not been possible. *Perspective* and *Triumph* were both positively associated with the experienced nostalgic effect of *Appreciation*, and both can relate semantically to the original nostalgic effect (*EEN*) statement, ‘It made me appreciate where my life is now because I have come so far.’ Therefore, the reasoning of *Perspective*, whether related to the self or general world view, and *Triumph*, which largely involved overcoming hardship, were viewed as notably positively due to these notions of redemption and perseverance.

Significant positive associations were found between *Reminder of People* and the reasoning categories of *Person or People*, *Performance*, *Trauma*, and *Pre-COVID Longing*. Many people who provided reasoning of their self-selected nostalgic piece of music based on *Performance* stated that they performed their chosen piece with certain people, and it reminded them of them, meaning their reasoning was coded into both categories. The same was the case for *Trauma*, which often related to loved ones who had passed away. For *Pre-COVID Longing*, participants expressed that they were separated from the people the music reminded them of and longed to see them again. Accordingly, the categories *Person or People, Performance, Trauma*, and *Pre-COVID Longing* were strongly overlapping, explaining why participants who provided reasoning relating to these categories were significantly more likely to agree with the *Reminder of People* nostalgic effect statement.

Finally, participants who agreed with the *Bittersweet* nostalgic effect statement were statistically significant more likely to provide nostalgic reasoning related to the coded categories of *Person or People*, *Perspective*, or *Trauma*. For the *Person or People* coded category, this can also be explained by the separation that exists between loved ones in the current climate, and therefore participants may have both happy memories of loved ones and feel sad that they are not with them. For the *Perspective* category, many indicated that they were reminded of a time in their life that was simpler than it is now, inducing similar bittersweet feelings of longing for the past and being stuck in the present pandemic. Lastly, as with the *Reminder of People* nostalgic effect, reasoning relating to *Trauma* may have induced mixed emotions due to both feelings of loss and being reminded of loved ones that were once in their lives.

### Research Question 2: Functions of Nostalgic Music as Emotion Regulation Strategies

The use of theemotion regulation strategies (*ERS-ACA)* scale in this study was well suited to studying strategies for nostalgic music listening. Using Confirmatory Factor Analysis on the 18 statements, all fit indices suggested that the model was a highly appropriate fit for the observed data. Each of the factors correlated with each other and showed high similarities with the factor loadings of the original study (Fancourt et al., 2019). Through analysis of the associations between nostalgic effects (*EEN)* and emotion regulation factors it was possible to determine which nostalgic effects were encompassed in each strategy.

For the *Avoidance* strategy, participants’ experience of nostalgia encompassed a focus on happier past times, and a disregard for sad difficult and past times, and bittersweet. This was expected, as Fancourt et al. (2019) stated that the *Avoidance* strategy involved avoiding or distracting from the negative. During the current crisis, reminiscing on previous times of hardship for some may have worsened the situation they were currently in if they felt that was also negative.

For *Approach*, participants experienced nostalgic effect comprised of a large focus on *Appreciation* and *Sad Difficult Times*. This strategy involved dealing with negative emotions directly (Fancourt et al., 2019). Participants’ therefore seemed to look at the difficult times in the past in the comparison to the present, in order to bring some perspective to their situations. Some of these participants may have experienced more severe hardship in the past that made the difficulties of the present seem insignificant. Alternatively, they may have experienced comparable hardship that they overcame, and reminded themselves of their own strength and perseverance.

For *Self-Development*, the only significant association was with the *Reminder of People*nostalgic effect. As this strategy encompassed items such as ‘It gives me a sense of purpose,’ ‘I feel more confident in myself,’ and ‘It boosts my self-esteem,’ it seems instinctual that these feelings would arise as a result of reminiscing about social interactions. For instance, the research outlined previously by Sedikides and Wildschut (2016), showed that nostalgia was seen to increase social connectedness, while simultaneously improving self-esteem, optimism, sense of purpose and meaningfulness in life. It is not clear whether the latter effects are a result of the former, or whether they co-occur. Nonetheless, nostalgia for some seems to be about reminding people of the social aspects of their life in order to establish meaning and purpose, even in times when such social aspects may be more distant.

The next stage of this research question focussed on possible associations between the emotion regulation strategy factors and coded variables of reasoning and emotional responses.

For coded reasoning, *Approach* raised a positive significant association with *Perspective*. This could suggest that those adopting an *Approach* regulation strategy with regards to nostalgic music are less likely to be nostalgic for a specific period of their former life, and instead, they might treat the past comparatively with their current situation. Reflecting on previous times of difficulty could provide strength to cope with the lockdown measures, and perspective on the hardships they could be faced with as a result of them. *Self-Development* was significantly positively associated with *Period of Life*. This suggests that contrary to the *Approach strategy*, those scoring highly in *Self-Development* may in fact immerse themselves in past periods of their life in order to learn from them and develop their present and future circumstances.

For the coded emotion analysis, each of the three emotion regulation factors presented the highest mean scores from the Positive emotion group. Factor scores from those coded into the Neutral group were the lowest. This supports previous research, suggesting that the use of any emotion regulation strategy in general, as opposed to not having a strategy, leads to the induction of positive emotion (Kashdan et al., 2014; Fancourt et al., 2019).

The second, and most fundamental part of this second research question looked at testing whether different emotion regulation strategies through listening to nostalgic music were associated with wellbeing (*SWEMWBS)*. The life impairment scale *WSAS* (Mundt et al., 2002) was incorporated here as a covariate inorder to control for the influence of impairment on wellbeing. A stepwise linear regression approach indicated that levels of life impairment accounted for a large amount of the variance for wellbeing, but of greater interest, the *Approach* strategy was positively associated with wellbeing and accounted for an additional 1% of its variance.

The first possibility is that those with higher levels of wellbeing were more likely to treat music-evoked nostalgia as an *Approach* emotion-regulation strategy, rather than simply dwelling in the past. The analysis of the emotion regulation (*ERS-ACA)* factors suggests a possible constraint of this study, whereby those with lower scores of wellbeing may have been altogether less likely to see nostalgia as an emotion regulation strategy, and as a result, the *ERS-ACA* scale would not have been able to capture this adequately. Participants who experience depression or had lower baseline wellbeing scores may have had a greater inclination to ruminate rather than resolve their issues, as explored by Garrido et al. (2016) in relation to individuals with depression and inclinations to ruminate and exacerbate negative thoughts through listening to sad music (Garrido and Schubert, 2015; Garrido et al., 2016). However, rumination was not captured in the questionnaire and was beyond the scope of this study. The second possible interpretation of the above finding is that higher wellbeing scores were a result of the treatment of music-evoked nostalgia as an *Approach* emotion regulation strategy in general.

Essentially, the *Approach* strategy is seemingly more applicable to the situation surrounding COVID-19 than ever. During such an uncertain time of hardship of unforeseen duration, forms of escapism associated with the *Avoidance* strategy may be the most unhelpful in relation to our wellbeing when it is not known how long one might be trying to escape for. Kashdan et al. (2014) highlight the potential for avoidance of personal anxieties as a strategy to enhance social anxiety in multiple contexts, which is highly applicable to the context of lockdown. Additionally, *Self-Development* is most difficult to grasp conceptually during lockdown periods whereby learning from the past to develop one’s present and future is difficult when it is not clear what the future holds. Therefore, it is logical that improvements to wellbeing were only seen with the *Approach* strategy.

As associations with nostalgic effects (*EEN)* suggest, the *Approach* strategy was focused around treating the past comparatively with the present; rather than escaping from the present by absorbing in periods past, participants reflected on aspects of the past that allowed them to feel more comfortable with the present circumstances. Whether this was through thinking of people they were separated from, or reflecting about sad times in the past, participants who used this strategy seemed to show higher levels of wellbeing. Previous research on terror management and nostalgia by Routledge et al. (2008) supports this finding, whereby reflection on the past can bring a sense of meaning to the present, allowing individuals to improve their perception of their current situation and counteract mortality concerns. This strategy allows for reflective comparison between past and present, and a means to cope by contrasting current hardship with both awareness of sad difficult past times and appreciating everything that has led up to the present.

## Limitations

Due to the nature of the lockdown restrictions, the questionnaire had to be distributed online and each participant responded remotely sometime after the first United Kingdom lockdown in 2020. Accordingly, we identified four main issues with regards to the questionnaire and data collection here, and recommendations for future research that were beyond the scope of this study.

The first issue was regarding the question of frequency of listening for nostalgic music. As this question was asked only in relation to nostalgic music, there was no other category of music to compare this with. Consequently, it is not possible to conclude that participants have engaged more during periods of lockdown than they otherwise would have, and that this finding that can be observed beyond this study.

The second issue was need for retrospective answers to questions regarding wellbeing and life impairment. This questionnaire was designed and distributed after the lockdown had been eased, and therefore participants were asked to think back to how they felt for the duration of the time between 23rd March and 23rd June. One problem with this is that their actual state of wellbeing and life impairment during this time may not have been reported accurately, as some may have not remembered how they felt several months before. Another problem with this is that their feelings may have not been consistent throughout this time. Depending on participants’ current mood, they may have reflected on this time more positively or more negatively. This leads on to another issue, in that the current mood of participants while they completed the questionnaire was not assessed, which could have had a large impact on their responses in general.

The third main issue seemed to be regarding the listening task portion of the questionnaire, which instructed participants to select one piece of music they had heard during lockdown which induced feelings of nostalgia. As can be seen from Supplementary Material 3, many participants selected albums, playlists, genres or entire eras of music. Therefore, this makes it difficult to be certain the participants went on to listen to a piece of music at all. Several participants’ responses were removed due to evident disregard for the procedure of the listening task. Some had stated they could not choose, and then provided fabricated responses for the rest of the questions associated with this listening task. Although some admitted they had done this in their comments at the end of the questionnaire, it could be possible that this occurred with many more that were unnoticed.

A fourth issue in this study was the order of questions presented in the listening task. Participants were asked for their reasoning behind why their selected piece of music induced feelings of nostalgia following their selection of it, rather than following listening to it. It may have been more useful to collect this information after listening to their chosen piece of music, so that the reasoning could be fresh in their minds, and most accurate. Many stated in their comments at the end that they wished they could go back and change their answers to some parts of the questionnaire having thought more about them, and answers to this reasoning question especially may have changed following the actual listening task.

Future research may better address the question of whether nostalgia was more prevalent through the first lockdown by assessing whether the extent they engaged with nostalgic music was more than they would prior to the COVID-19 outbreak. Additionally, it may be appropriate to compare this with engaging with new or undiscovered music. Furthermore, where previous research has often referred to nostalgic music as beingfrom a certain era or within a timeframe (see Yu-Cheong Yeung, 2020), this study acknowledged that nostalgic music is entirely person- and context-dependent. A consequence of this was that answers were extremely widespread, ranging from songs to entire genres. We encourage further studies to also consider the individualistic nature of nostalgic music, but make attempts to better contain and limit responses, in order to study patterns within the music selection process, and the music itself. Future research may also wish to explore the impact of interindividual differences related to social demographics, which was not a focus of our study. Research of interest could be the impact that differencesin gender, age, level of musicianship, work status, or household may have on the nostalgic music listening, emotion regulation strategies and resulting levels of wellbeing during times of crisis.

## Conclusion

This study aimed to investigate individual experience of music-evoked nostalgia in relation to levels of wellbeing during the COVID-19 crisis in the United Kingdom. We explored emotional content, functions, and effects of music-evoked nostalgia during the enforcement of lockdown restrictions. Overall, it seems that there is an association between the use of nostalgic music as an *Approach* emotion regulation strategy and levels of wellbeing. This suggested that the extent one considers nostalgic music as an *Approach* emotion regulation strategy influences wellbeing; whether through acknowledgment and reflecting on sad difficult times, and a sense of appreciation, whether this is appreciation for anything positive in the present circumstances, or appreciation for anything that has led up to present circumstances. In general, this research contributes evidence of the benefits in engaging with nostalgic music to regulate emotions during times of uncertainty, and why reflecting on the past to bring perspective to the present, or the *Approach* strategy, is linked to a positive state of wellbeing. This study was not able to identify psychological issues as a result of nostalgic music listening behaviour, however, we encourage future research in this area. This would certainly be possible providing researchers acknowledge that nostalgia is a person- and context- dependent phenomenon and treat it accordingly.

## Data Availability Statement

The raw data supporting the conclusions of this article will be made available by the authors, without undue reservation.

## Ethics Statement

The studies involving human participants were reviewed and approved by Arts and Humanities Ethics Committee, University of York. The patients/participants provided their written informed consent to participate in this study.

## Author Contributions

HG designed and conducted the study, analysed the data, and also created a first draft of the manuscript. HE contributed to analysing the data, developed an overall narrative for the presentation of results, and revised the manuscript. Both authors contributed to the article and approved the submitted version.

## Conflict of Interest

The authors declare that the research was conducted in the absence of any commercial or financial relationships that could be construed as a potential conflict of interest.
