# Supplementary material for: Music-Evoked Nostalgia and Wellbeing During the United Kingdom COVID-19 Pandemic: Content, Subjective Effects, and Function
Source: Front Psychol. 2021 Mar 22;12:647891. doi: 10.3389/fpsyg.2021.647891 (PMC8019926; doi:10.3389/fpsyg.2021.647891)
Supplement: Supplementary file 1 [file Table_1.DOCX]

S1: Online Qualtrics Survey

An exploratory analysis of nostalgic music listening during the COVID-19 pandemic

(Greyed font not shown to participants)

*Nostalgic music listening and wellbeing during the COVID-19 pandemic in the UK*
Thank you for agreeing to partake in this study. Before proceeding further, please read through the following information:
*General Overview and Background***:** The proposed research aims to investigate music, nostalgia, emotion regulation and wellbeing, alongside subjective experiences of the UK lockdown.
This research is being carried out by MA Music Psychology student Hannah Gibbs (hjg516@york.ac.uk), supervised by Dr Hauke Egermann (hauke.egermann@york.ac.uk), and is based at the University of York, part of the York Music Psychology Group (YMPG). Ethics approval has been granted by the University of York ethics committee, with Dr Tom Collins (tom.collins@york.ac.uk) as the most appropriate port of call on the ethics committee, should you have any questions or concerns.
Disclaimer: You will be asked about your current state of wellbeing, and your feelings towards a piece of music that may evoke specific memories. In some cases, participants may find this distressing. For the purposes of this research, honest responses are necessary in order for the data gathered to maintain reliability and internal consistency. Please do not hesitate to email any of the contacts provided if you do experience any distress or have any concerns. 
If you are not residing in the UK, apologies, but this study is not relevant for you. This is because every country has dealt with the pandemic in a variety of ways.

What do I need to do? Complete the questionnaire in full, which may include a section that requires you to listen to your own selected piece of music, depending on your responses. For the music listening, you will need good quality headphones or loudspeakers and access to Youtube, Spotify, or whatever listening platform you choose. Ideally, you should find some privacy, away from interfering noise or distractions.

How long will the questionnaire take to complete? It should take no more than 15 minutes.

Who will the study be with? Yourself and your device.

For more information on data processing and GDPR compliance, [please click here.](https://drive.google.com/file/d/1AZ8ZWkRJMFW2nysXkYvaO-hD8H_FmR2J/view?usp=sharing)
<https://drive.google.com/file/d/1AZ8ZWkRJMFW2nysXkYvaO-hD8H_FmR2J/view?usp=sharing>
 Please tick below if you are happy to proceed:

Yes, I consent to my anonymised data being processed for the purposes outlined

Are you currently based in the UK, or have lived in the UK throughout the majority of the pandemic?

- Yes
- No

Where in the UK are you residing?

- England
- Wales
- Scotland
- Northern Ireland
- Other (British Islands)

Consider your situation from the start of the official national lockdown (23rd March) up until the publication date of this survey (June 23rd). How has the pandemic impaired the following for you over the last 3 months:
If you have recognised a positive change rather than impairment, please select 'Not at all'.

(Impairment scale adapted from *WSAS*)

|  | Not at all | Slightly | Moderately | Definitely | | Markedly | Very Severely |
| --- | --- | --- | --- | --- | --- | --- | --- |
| Finances |  |  |  |  |  | |  |
| Ability to work |  |  |  |  |  | |  |
| Ability to study |  |  |  |  |  | |  |
| General mental or physical health |  |  |  |  |  | |  |
| Home management (cleaning, tidying, shopping, cooking, looking after home or children, paying bills) |  |  |  |  |  | |  |
| Engagement with your usual private leisure activities (done alone) |  |  |  |  |  | |  |
| Engagement with your usual social leisure activities |  |  |  |  |  | |  |
| Ability to form and/or maintain close relationships (including those I live with) |  |  |  |  |  | |  |

Below are some statements about feelings and thoughts. Please give tick the box that best describes your experience of each over the last 3 months.
(*Short Warwick-Edinburgh Mental Well-being Scale*)

|  | None of the time | Rarely | Some of the time | Often | | All of the time |
| --- | --- | --- | --- | --- | --- | --- |
| I’ve been feeling optimistic about the future |  |  |  |  |  | |
| I’ve been feeling useful |  |  |  |  |  | |
| I’ve been feeling relaxed |  |  |  |  |  | |
| I’ve been dealing with problems well |  |  |  |  |  | |
| I’ve been thinking clearly |  |  |  |  |  | |
| I’ve been feeling close to other people |  |  |  |  |  | |
| I’ve been able to make up my own mind about things |  |  |  |  |  | |

The emotion of nostalgia is defined by the The New Oxford Dictionary of English (1998) as “a sentimental longing or wistful affection for the past” (p. 1266). Nostalgia can make you feel both good and bad, whether you mourn the past or look backwards to remind you of the happiness you have felt in your life.

 “The nostalgizer, then, is presumed to feel negatively for a bygone way of life, for the passing of treasured moments, and for the current absence of persons significant to them. At the same time, the nostalgizer feels positively for having had the opportunity to share defining life events with those significant others.” (Sedikides & Wildschut, 2016)

Nostalgic music is defined as music which gives you a feelings of nostalgia, that is, feelings of sentimental longing for the past.  The next series of questions relate to your experience of nostalgic music. This could be music from specific times of your life, associated with certain people, or music you listened to when you were younger. It may be bittersweet, positive or negative, or a mixture of the two.

(Fancourt et al., *ERS-ACA* scale)

Regardless of why, how much do you agree with the statements below? When engaging in nostalgic music listening...

|  | Strongly disagree | Somewhat disagree | Neither agree nor disagree | Somewhat agree | Strongly agree |
| --- | --- | --- | --- | --- | --- |
| I can block out any unwanted thoughts or feelings |  |  |  |  |  |
| I can shake off any anxieties in my life |  |  |  |  |  |
| I feel I am in my own little bubble, away from ordinary worries |  |  |  |  |  |
| It helps me forget about my worries |  |  |  |  |  |
| It helps me to disengage from things that are bothering me |  |  |  |  |  |
| It makes me feel detached from negative things in my life |  |  |  |  |  |
| It redirects my attention so I forget unwanted thoughts and feelings |  |  |  |  |  |
| I can contemplate what is going on in my life with a clear mind |  |  |  |  |  |
| It helps me refocus on what matters in my life |  |  |  |  |  |
| It helps me to come to terms with my own emotions |  |  |  |  |  |
| It helps me to put worries or problems I have in perspective |  |  |  |  |  |
| It helps me to understand my own feelings on things that are on my mind |  |  |  |  |  |
| It makes me reflect on my emotions |  |  |  |  |  |
| I feel more confident in myself |  |  |  |  |  |
| It boosts my self-esteem |  |  |  |  |  |
| It gives me a sense of purpose |  |  |  |  |  |
| It makes me feel stronger in myself |  |  |  |  |  |
| It reaffirms my identity |  |  |  |  |  |

How often have you found yourself listening to nostalgic music, either intentionally or coincidentally, over the last 3 months?

- Never
- Less than once a month
- At least once a month
- At least once a fortnight
- At least once a week
- At least once a day

Please provide details of a piece of music or song you have heard in the last 3 months that made you feel nostalgic. You may either provide artist and title (and a particular version if necessary) or provide a link.

________________________________________________________________

Can you think of a particular reason that this piece makes you feel nostalgic?
Examples could include an event, situation, place or person.

________________________________________________________________

Please listen to the piece of music you have selected prior to continuing with this survey.

- Yes, I have listened to my chosen piece

How do you feel having just listened to the song? Please try and use one word to summarise.

________________________________________________________________

The following items relate to how you feel after listening to your selected piece. Please rate how much you agree with each statement.

(Taken from Garrido (2016) *Experienced Effects of Nostalgia* (*EEN*)*)*

|  | Strongly disagree | Disagree moderately | Disagree a little | Neither agree nor disagree | Agree a little | Agree moderately | Strongly agree |
| --- | --- | --- | --- | --- | --- | --- | --- |
| It made me happy to think about happy times in the past. |  |  |  |  |  |  |  |
| It made me sad because those happy times I had in the past are gone. |  |  |  |  |  |  |  |
| It made me appreciate where my life is now because I have come so far. |  |  |  |  |  |  |  |
| It made me sad remembering difficult times in the past. |  |  |  |  |  |  |  |
| It reminded me of people that I used to see more often and I enjoyed thinking about them and feeling more connected to them. |  |  |  |  |  |  |  |
| The experience was bittersweet, somewhat happy and somewhat sad. |  |  |  |  |  |  |  |

Here are a number of personality traits that may or may not apply to you. Please select an option for each statement to indicate the extent to which you agree or disagree with that statement. You should rate the extent to which the pair of traits applies to you, even if one characteristic applies more strongly than the other.

(*Ten-item Personality Inventory,* Gosling et al., (2003))

|  | Disagree strongly | Disagree moderately | Disagree a little | Neither agree nor disagree | Agree a little | Agree moderately | Agree strongly |
| --- | --- | --- | --- | --- | --- | --- | --- |
| Extraverted, enthusiastic. |  |  |  |  |  |  |  |
| Critical, quarrelsome. |  |  |  |  |  |  |  |
| Dependable, self-disciplined. |  |  |  |  |  |  |  |
| Anxious, easily upset. |  |  |  |  |  |  |  |
| Open to new experiences, complex |  |  |  |  |  |  |  |
| Reserved, quiet. |  |  |  |  |  |  |  |
| Sympathetic, warm. |  |  |  |  |  |  |  |
| Disorganized, careless. |  |  |  |  |  |  |  |
| Calm, emotionally stable. |  |  |  |  |  |  |  |
| Conventional, uncreative. |  |  |  |  |  |  |  |

How old are you (in years)?

________________________________________________________________

What is your gender?

- Male
- Female
- Prefer to self-describe ________________________________________________
- Prefer not to say

What best describes your work status during the majority of lockdown?

- Student
- Professional
- Unemployed
- Furloughed, or out of work temporarily
- Retired
- Disabled
- Other ________________________________________________

Are you a musician? If so, which would best describe your level of musicianship?
  
Grade standards have been given to provide a more objective scale, but you do not have to have completed any grades to consider yourself a musician. They are intended as a measure of the level of competence and understanding you have of music, whether this is relevant to a particular instrument, voice, or music production.
 If you are unsure what these standards relate to, please see [this guidance](https://www.nidirect.gov.uk/articles/qualifications-what-different-levels-mean).
<https://www.nidirect.gov.uk/articles/qualifications-what-different-levels-mean>

- No
- Beginner (up to grade 1 standard)
- Amateur (up to grade 3 standard)
- Intermediate (up to grade 5 standard)
- Higher level (up to grade 8 standard)
- University student or semi-professional
- Professional

Which of the following best describes your situation?

- The UK has been my permanent country of residence for the majority of my life
- I was not raised in the UK, but I have lived here for 4 years or more and consider it to be my permanent country of residence
- I came to live in the UK within the last 4 years and I plan on staying here for the foreseeable future
- I came to live in the UK within the last 4 years and I plan on returning to my home country
- None of the above (please self-describe) ________________________________________________

Which best describes your household situation during the majority of lockdown? If you live with others please elaborate on who they are (friends, partner, relatives, etc).

- I live alone
- I live with others ________________________________________________

How well were you able to focus throughout this survey and give each question your full attention?

- Not well at all
- Only a little
- Moderately
- Quite well
- Extremely well

Please provide any further comments here.

________________________________________________________________
