# Supplementary material for: Music-Evoked Nostalgia and Wellbeing During the United Kingdom COVID-19 Pandemic: Content, Subjective Effects, and Function
Source: Front Psychol. 2021 Mar 22;12:647891. doi: 10.3389/fpsyg.2021.647891 (PMC8019926; doi:10.3389/fpsyg.2021.647891)
Supplement: Supplementary file 2 [file Table_2.DOCX]

S2: ERS-ACA Factor Loadings

| Table for Section 3.2.1. Factor loadings for *ERS-ACA* factors | | | | | | | |
| --- | --- | --- | --- | --- | --- | --- | --- |
|  | | | | | | 95% Confidence Interval | |
| Factor | Indicator | Estimate | Std. Error | z-value | p | Lower | Upper |
| *Avoidance* | I can block out any unwanted thoughts or feelings | 0.780 | 0.039 | 20.073 | < .001 | 0.704 | 0.856 |
|  | I can shake off any anxieties in my life | 0.726 | 0.038 | 19.251 | < .001 | 0.652 | 0.800 |
|  | I feel I am in my own little bubble, away from ordinary worries | 0.584 | 0.035 | 16.608 | < .001 | 0.515 | 0.653 |
|  | It helps me forget about my worries | 0.735 | 0.037 | 20.028 | < .001 | 0.663 | 0.807 |
|  | It helps me to disengage from things that are bothering me | 0.621 | 0.033 | 18.657 | < .001 | 0.556 | 0.686 |
|  | It makes me feel detached from negative things in my life | 0.719 | 0.038 | 19.134 | < .001 | 0.645 | 0.792 |
|  | It redirects my attention so I forget unwanted thoughts and feelings | 0.723 | 0.036 | 19.877 | < .001 | 0.652 | 0.794 |
| *Approach* | I can contemplate what is going on in my life with a clear mind | 0.642 | 0.037 | 17.529 | < .001 | 0.570 | 0.714 |
|  | It helps me refocus on what matters in my life | 0.696 | 0.037 | 18.981 | < .001 | 0.624 | 0.768 |
|  | It helps me to come to terms with my own emotions | 0.634 | 0.035 | 18.165 | < .001 | 0.566 | 0.703 |
|  | It helps me to put worries or problems I have in perspective | 0.659 | 0.038 | 17.326 | < .001 | 0.584 | 0.733 |
|  | It helps me to understand my own feelings on things that are on my mind | 0.689 | 0.035 | 19.694 | < .001 | 0.620 | 0.757 |
|  | It makes me reflect on my emotions | 0.448 | 0.036 | 12.469 | < .001 | 0.377 | 0.518 |
| *Self-Development* | I feel more confident in myself | 0.760 | 0.036 | 21.019 | < .001 | 0.689 | 0.831 |
|  | It boosts my self-esteem | 0.737 | 0.039 | 19.107 | < .001 | 0.661 | 0.812 |
|  | It gives me a sense of purpose | 0.593 | 0.038 | 15.605 | < .001 | 0.519 | 0.668 |
|  | It makes me feel stronger in myself | 0.758 | 0.035 | 21.414 | < .001 | 0.688 | 0.827 |
|  | It reaffirms my identity | 0.628 | 0.041 | 15.516 | < .001 | 0.549 | 0.708 |
| Note: *N*=570 | | | | | | | |
