# Supplementary material for: Music-Evoked Nostalgia and Wellbeing During the United Kingdom COVID-19 Pandemic: Content, Subjective Effects, and Function
Source: Front Psychol. 2021 Mar 22;12:647891. doi: 10.3389/fpsyg.2021.647891 (PMC8019926; doi:10.3389/fpsyg.2021.647891)
Supplement: Supplementary file 3 [file Table_3.DOCX]

S3: Participants’ Self-Selected Nostalgia Music

Below is a list of all self-selected nostalgic music, given in response to the question of ‘Please provide details of a piece of music or song you have heard in the last 3 months that made you feel nostalgic. You may either provide artist and title (and a particular version if necessary) or provide a link.’
Some participants chose to select more than one song, an album, a playlist, or a radio station. 568 responses are presented, as several provided links that were expired or broken.

| Huun-Huur-Tu - Orphan's Lament |
| --- |
| Dukas - The Sorcerer's Apprentice |
| Nina Hagen - Eisern Union |
| Rui Da Silva - Touch Me |
| Build Me Up Buttercup - The Foundations |
| Sing for Absolution - Muse |
| Those Were The Days - Mary Hopkin |
| Arctic Monkeys- Do I Wanna Know |
| Aphex Twin - Avril 14th |
| Chris Rea - Wired to the Moon |
| iamamiwhoami - FOUNTAIN |
| Shearwater - St Mary’s Walk |
| Champagne Supernova - Oasis |
| Ryan Davies - Pan fo'r nos yn hir |
| Anything by Nick Cave |
| My Chemical Romance - Black Parade |
| Stardust - Music Sounds Better With You |
| Talking Heads - 77 (album) |
| DJ Luck & McNeat - With a Little Bit of Luck |
| The Strokes - Is This It |
| New model army thunder and consolation (album) |
| Kim Larsen - Sammen Og Hver For Sig |
| Pale - Come Back to Me |
| Stars - No-one is Lost |
| George Gershwin - Summertime |
| Space oddity - David Bowie |
| Woodkid - Run Boy Run |
| King Tubby – Zion Gate Dub |
| Duke Browner - Crying Over You |
| Beach Boys - I Can Hear Music |
| Rolling Stones – It’s All Over Now |
| Van Halen – Romeo’s Delight |
| Bruce Springsteen – Sad Eyes |
| Deee-lite - Groove Is In The Heart |
| Eve Selis- See Me With Your Heart |
| The National - England |
| 70s music: Eagles, James Taylor, Carol King etc. |
| E.Coli (feat. Earl Sweatshirt), The Alchemist, Earl Sweatshirt |
| Kate Rusby (arr. Clements, performed by VOCES8) - Underneath the Stars |
| Vera Lynn - We’ll Meet Again |
| Mudhoney - Touch Me I'm Sick |
| Andrew Lloyd Webber - Pie Jesu |
| Bob Dylan - Like a Rolling Stone |
| Mcfly - All About You |
| Mario - Let Me Love You |
| Odyssey - Native New Yorker |
| Broken Social Scene - Swimmers |
| Led Zeppelin - The Rover |
| The OC soundtrack - More Bounce In Califorinia |
| Bittersweet Symphony - The Verve |
| Mostly Russian music because I come from Russia and don’t listen to English music. Some songs remind me of my teenage years and some are more recent. The most recent one is a new song I heard when visiting friends abroad the week before lockdown: Khrystyna Soloviy - Trymai |
| Nirvana MTV Unplugged in New York |
| Nick Drake - Which Will |
| David Bowie – Absolute Beginners |
| Fleetwood Mac - Gypsy |
| The Chris Barber Traditional Jazz Band |
| The The - This Is the Day |
| Queen - Greatest Hits (album) |
| Tchaikovsky - The Seasons, June, Barcarolle Op. 37 no. 6 |
| Ben Howard - Grow |
| The Kinks - Waterloo Sunset |
| Free - All Right Now |
| Gorrilaz - O Green World |
| New Order |
| The Delgados - Peloton |
| The Wanted - Rocket |
| Love Divine (Blaenwern) |
| The Mamas & The Papas - California Dreaming |
| Radiohead - Lotus Flower (Jaques Green remix) |
| Dusty Springfield & Pet Shop Boys - Nothing Has Been Proved |
| Bill Medley & Jennifer Warnes - I’ve Had the Time of My Life |
| Elliott Smith - Angeles |
| Joni Mitchell - The Urge for Going |
| The Cocteau Twins |
| Moody Blues- Nights in White Satin |
| Aram Khachaturian - Spartacus Adagio |
| Haircut 100 - Fantastic Day |
| Jamelia - Superstar |
| Rachmaninov - Piano Concerto no. 2 |
| Ralph McTell - Barges |
| Mozart (Vladimir Horowitz, Orchestra of La Scala) - Piano Concerto no. 23, 2^nd^ Mov |
| Millencolin - No Cigar |
| Ferde Grofe - Grand Canyon Suite |
| Mozart - Requiem |
| Bic Runga - Drive |
| Purcell (Jessye Norman) - When I Am Laid in Earth |
| Faure - Requiem |
| Massive Attack - Unfinished Sympathy |
| The Bodysnatchers - Let's do Rock Steady |
| Welsh National Anthem |
| Li Di (이적) - Don’t worry, my dear (걱정말아요 그대) |
| Major Lazer - Get Free |
| Chrono Cross OST - Star Stealing Girl |
| The Slits - Typical Girls |
| Karl Jenkins - Requiem |
| The Clash - London Calling |
| Herbert Sumsion - They That Go Down to the Sea in Ships |
| Michael Nyman - In Re Don Giovanni |
| The B52s - Rock Lobster |
| Duke - So in Love with You (full intention mix) |
| Fiorella Mannoia - Il Cielo d'Irlanda |
| Rolling Stones concerts on TV |
| Orbital - Belfast |
| Bread - Guitar Man |
| Matt Munro - Walk Away |
| Roger Bart - Go the Distance (from Disney's Hercules soundtrack) |
| Pink Floyd |
| Louis Armstrong - Wonderful World |
| Panic! At The Disco - Hallelujah |
| Carly Rae Jepson - I Really Like You |
| Charlie Byrd and Stan Getz - Jazz Samba |
| Dvorak - Serenade for Strings |
| Red Hot Chili Peppers - Wet Sand |
| The Clash - Rudie Can’t Fail |
| Surfjan Stevens - Chicago |
| Tom T Hall - Old Dogs; Children; Watermelon Wine |
| John Lennon - Imagine |
| Erica Freas – Young (LP) |
| Michael Collins (Irish trad.) - My Lagan Love |
| Tori Amos - Tear in Your Hand |
| Glenn Miller - Nightingale Sang In Berkeley Square |
| Eels - Novacaine for the Soul |
| Elina - Here with Me |
| Bloc Party - The Pioneers |
| The Verve - Bittersweet Symphony |
| Sibelius - Symphony No. 1 |
| Kate Bush - Hounds of Love |
| A lot of 90's music |
| Yes - Wondrous Stories |
| Nine Inch Nails - We're in This Together |
| The Beach Boys - Good Vibrations |
| The Clash - London Calling |
| Steps - 5, 6, 7, 8 |
| Breach - Fatherless |
| HNNY - Nothing (Original Mix) |
| Nine Inch Nails - Terrible Lie |
| Simple Minds - Alive and Kicking |
| Clever Girl - Elm |
| Bee Gees - You Win Again |
| Vera Lynn - We'll Meet Again |
| Fleetwood Mac - Dreams |
| Milton Nascimento - Travessia |
| David Bowie - Wild is the Wind |
| Gustav Holst - Ave María |
| Warung Pojok (Tradition Indonesian Gamelan, Ndangdhut) |
| Tim Buckley - Song to the Siren |
| Mac Demarco - Preoccupied |
| The Rhythm Method - Something for the Weekend |
| Deerhunter - Microcastle (whole album) |
| Athlete - Wires; Danny L Harle - Broken Flowers; Red Hot Chilli Peppers - Can't Stop |
| Corelli - Violin Sonata Op 6, C Major |
| Debussy - String Quartet |
| Rachmaninov - Symphony no. 2 |
| Annie Lennox |
| Marry The Night - Lady Gaga |
| Sufjan Stevens - Futile Devices |
| Gerald Finzi - Eclogue for Piano and Strings |
| A-ha – Take On Me |
| John Williams - Harry Potter and the Philosophers Stone Soundtrack |
| Joe Goddard - Music is the Answer |
| Miles Davis & John Coltrane - Oleo |
| Van Morrison - Moondance |
| Paramore - Riot; Brand New Eyes (albums) |
| Boris Vian - Le Déserteur |
| Fred Astaire - The Way You Look Tonight |
| Kate Tempest - People's Faces |
| Tame Impala - Half Full Glass Of Wine |
| Weyes Blood - Wild Time |
| Norah Jones - Come Away With Me |
| The 1975; Mahler Symphonies; Bruch - Violin Concerto |
| BB King |
| Mount Kimbie - Made to Stray |
| Elgar - Nimrod from 'Enigma Variations' |
| Stevie Wonder - Another Star |
| Flamingods - Majesty (album) |
| Gym Class Heroes - Clothes Off!; Bloc Party - Mercury |
| Oasis - Who Feels Love |
| The Lightening Seeds - Life of Riley |
| S.O.S Band - Have it Your Way |
| Leonard Cohen - Suzanne; Like a Bird on a Wire |
| The Beatles - Here Comes the Sun |
| Liverpool Scene - Percy Paslow's Hampster farm |
| Les Swingle Singers - Fugue en Re Mineur (from Jazz Sebastien Bach) |
| Beth Nielson Chapman - I Find Your Love |
| Andrea Begley- Latch |
| Gerry and The Pacemakers - You’ll Never Walk Alone |
| Bach - Mass in B minor |
| Lou Reed - Sad Song |
| Robert Fripp - North Star |
| Richard Strauss, Jessye Norman - Four Last Songs |
| Radiohead - My Iron Lung (Glastonbury 1997) |
| Nicki Minaj |
| Appleton - Don't Worry |
| Christina Perri - A Thousand Years |
| Sonny Rollins - Wail March (from Blue Note Vol. 2) |
| Blockbuster |
| Katharine McPhee - She Used to be Mine (from The Waitress) |
| In Christ Alone (Hymn) |
| Coldplay |
| Blink 182 - Asthenia |
| 落日飞车 - My Jinji |
| Four Tet - Lush |
| Vivaldi - Gloria |
| She Moved Through the Fair (Traditional Irish folk song) |
| The Dark Sidd of the Moon |
| Duran Duran - Rio (album); Seven and the Ragged Tiger (album) |
| Design for Life - Manic Street Preachers |
| Calon Lân (Welsh Traditional) |
| My 80’s playlist |
| Queens of the Stone Age - No-one Knows |
| Rachmaninov - Piano Concerto no. 2 |
| Mozart - Marriage of Figaro, Act 2 finale, ‘Conoscete, signor Figaro’ |
| Jimmy Eat World - The Middle; Jeff Buckley - Hallelujah; Feeder - Just a Day |
| The Waterboys - This is the Sea |
| Natasha Beddingfield - Unwritten |
| Vistas - Medicine; John Brion - Strings That Tie to You; Radiohead - reminder; Muse - Shine |
| The Carpenters - Yesterday Once More |
| The Killers - Mr Brightside |
| East India Youth - Dripping Down |
| McFly- Room on the Third Floor |
| The Offspring - Pay The Man |
| Britney Spears - Toxic |
| Shawn Mendes - Stitches |
| Richi e Poveri - Sarà Perché Ti Amo |
| Led Zeppelin - The Lemon Song |
| Rage Against the Machine - Zabriskie Point (album) |
| Kevin Rudolf ft. Lil Wayne - Let it Rock |
| Astrid S - Hurts So Good |
| Eric Whitacre - Glow |
| PC Music, Sophie, Danny L Harle; Yamaneko - Pixel Wave Embrace (album), Ryuichi Sakamoto |
| Tomte - New York |
| Lorde - Green light |
| Yves Tumor - Limerence |
| Kate Bush |
| William Harris - Bring Us, O Lord God |
| Chopin - Nocturne no. 13 |
| Reel Big Fish - Take On Me |
| Foo Fighters |
| Andrew Lloyd Webber - Potiphar (from Joseph and the Amazing Technicolour Dreamcoat) |
| Bob Marley Trenchtown Rock |
| Backstreet Boys- I want it that way |
| Four Tet - Lush |
| Lost Frequencies - Are You With Me |
| ABBA |
| Glenn Miller - String of Pearls |
| Faure - Cantique de Jean Racine |
| Ravel - Daphnis & Chloe |
| Mozart - Sinfonia Concertante for Violin and VIola |
| Tchaikovsky - Symphony no. 6 |
| Smetana - Ma Vlast |
| Finlandia - Sibelius |
| Ray Parker Jr - Ghostbusters |
| Kate Bush - Army Dreamer |
| Elgar - Symphony no. 2 |
| Leonard Cohen - Hallelujah |
| Anonymous 4 - A Mass for the End of Time |
| Erykah Badu - Drama |
| Kate Bush - Wuthering Heights; Woman’s work; Running Up That Hill |
| Blink-182 - I Miss You |
| Adam De Sorgo - A Mighty Fortress Is Our God (Arranged for Oboes) |
| Gerry Rafferty - Baker Street |
| R&J Stone - We Do It |
| Poker Face - Lady Gaga |
| Wheatus - Teenage Dirtbag |
| Ides of March - Vehicle |
| Bon Iver - re:Stacks |
| Bowerbirds - Northern Lights |
| Stone Roses - Made of Stone |
| Metallica - So What (cover) |
| Lots of 80s music; Take That |
| Jack Johnson - Better Together |
| Gene - Your Love, It Lies |
| Meatloaf - I Would Do Anything For Love |
| ELO - Mr Blue Sky |
| Elton John - Captain Fantastic and the Brown Dirt Cowboys (album) |
| Counting Crows - Round Here |
| Keith Michel - Captain Beaky |
| Zaz |
| Gala - Freed From Desire |
| Wham - Bad Boys |
| Ray Davies - One More Time |
| Buddy Holly - Rocking Around with Ollie Vee |
| Hazel O'Connor - Will you |
| Where Do the Children Play - Cat Stevens |
| The Adventures- Broken Land |
| Nora Jones - come away with me |
| Most Sunday night's broadcasts by radio station Wave 105, program called Teenage Kicks |
| Fall Out Boy - Sugar, we're going down |
| Pearl Jam - Even Flow |
| Bob Seeger and the Silver Bullet Band - Hollywood Nights |
| David Bowie - Sound and Vision |
| Dolly Parton and Kenny Rogers - Islands in the Stream |
| Things can only get better - D:Ream |
| Aretha Franklin - Think |
| Wham! - Wake Me Up Before You Go Go |
| Vera Lynn -White Cliffs of Dover |
| Roxette - Joyride |
| Janis Joplin - Little Girl Blue |
| Bryan Adams - Summer of 69 |
| Zero 7 - Simple Things (album) |
| Rise up from Hamilton; Halestorm – Heres’ To Us; Deep Blue Something - Breakfast at Tiffany's |
| Stockhausen - Mittwoch Aus Licht |
| John Illsley - Never Told a Soul |
| Genesis - Invisible Touch |
| Gary Moore - Parisienne Walkways |
| The Cult - Fire Woman |
| Sandi Thom - I Wish I Was a Punk Rocker; Simon and Garfunkel - The Boxer; and Israel Kamakawiwo’ole - Somewhere over the rainbow |
| Queen - I Want to Break Free |
| 80s soul music |
| Elo – Ticket to the Moon |
| Django Reinhardt |
| Apocalyptica |
| Train - Drops of Jupiter |
| Rage Against the Machine - Know Your Enemy |
| Duran Duran - Save a Prayer |
| My 80s playlist |
| Madness - It Must Be Love |
| Pink Floyd - Shine on Crazy Diamond |
| Sum 41 - In Too Deep |
| Nirvana - Man Who Stole the World |
| Big Country - In a Big Country |
| Love Affair - Everlasting Love |
| Mary Hopkin - Those Were the Days |
| The Darkness - I Believe in a Thing Called Love |
| Olly Murs - Dear Darlin; Dido - White Flag |
| Eva Denis - I Wish I Could Cry My Heart Out |
| Gram Parsons - Sin City |
| Lemar - Neverending Story |
| Bon Jovi - Living on a prayer |
| The Carnival of the Animals; Danse Macabre; Any Classical, Jazz or Swing music; cheesy pop hits from the 2000s. |
| Mike Oldfield - Tubular Bells |
| Nirvana - Come as you are |
| The Shadows |
| U2 - Sunday Bloody Sunday |
| Queen - The Show Must Go On |
| Pharrell Williams - Happy |
| Patsy Cline, Foo Fighters and Kiss |
| Crowded House -Weather With You |
| Dire Straits - Brothers in Arms |
| Roxy Music - For Your Pleasure (album) |
| No songs in particular, but have listened to long forgotten playlists from many years ago |
| Whitney Houston - One Moment in Time |
| Queen and musicals I was part of |
| Red Hot Chilli Peppers - Under the Bridge |
| Queen - Greatest Hits (albums) |
| Meatloaf - Bat Out of Hell |
| Tom Lehrer - I Got it From Agnes, |
| Blink 182 - What’s My Age Again |
| Fragma - Toca's Miracle |
| Celine Dion - You Gave Me Strength |
| Prince - Diamonds and Pearls, Gold; DJ Jazzy Jeff & Fresh Prince - Summertime |
| Erasure - Drama |
| The Cult - Sanctuary |
| Bay City Rollers - Bye Bye Baby |
| Genesis - Afterglow |
| Michael Ball & Alfie Boe |
| Sia - Chandelier |
| Tchaikovsky - 1812 Overture |
| My Chemical Romance |
| Elastica - Connection |
| Crowded House - Distant Sun |
| Pink Floyd - Wish You Were Here (album) |
| Public Enemy - Black Steel in the Hour if Chaos |
| Dobie Gray - Drift Away |
| James - Sit Down |
| Flyleaf - Broken Wings |
| McFly - Obviously |
| John Legend - All of Me |
| Vera Lynn - We'll Meet Again |
| Sound of Silence - Dave Draiman |
| Pink floyd - Fearless |
| Christopher Cross - Arthur's Theme |
| Show of Hands - Bristol Slaver |
| Papa Roach - Last Resort |
| Bon Jovi |
| Output from Radio Dismuke which covers music from 1920s and 1930s |
| Tina Turner - Simply the Best |
| Queen - Somebody to Love |
| Goldfinger - Counting the Days |
| Faithless - Insomnia |
| Spandau Ballet - Through the Barricades |
| Sugababes - Too Lost in You |
| Faith Hill - There You’ll Be |
| Rondo Veneziano - Notturno in Gondola (from Not Quite Jerusalem) |
| Anything by Ferocious Dog or Gaz Brookfield |
| Queen - Greatest Hits (album) |
| Tim Minchin - Rock 'n' Roll Nerd |
| Måns Zelmerlöw - Heroes (Eurovision Song Contest winner) |
| Powderfinger - My Happiness |
| N-trance - Set You Free |
| Karma Chameleon Boy George |
| David Bowie - Let's Dance |
| Rammstein - Mein Herz Brennt |
| Ash - Girl From Mars |
| Evenanescence - My Immortal |
| Alfie Boe |
| ABBA - Andante Andante |
| Carly Simon - Embrace Me You Child |
| Type O Negative - Bloody Kisses (album) |
| Vera Lynn - We’ll Meet Again |
| Led Zeppelin - Stairway to Heaven |
| Adam Ant - Wonderful |
| Richard Marx - Hazard |
| Fear Factory - Linchpin |
| Mary Chapin Carpenter - Jubilee |
| iLife on Mars by David Bowie |
| The Beatles - I am the Walrus |
| The Mamas & The Papas - Dream a Little Dream |
| Disturbed - Sound of Silence (cover) |
| Queen - Innuendo |
| Temple Of Love on Planet Rock (Radio) |
| Thomas Newman - Shawshank Prison (Stoic Theme) |
| Manic Street Preachers; S Club 7; Taylor Swift |
| The Shadows - Apache |
| Leonard Cohen - Going Home |
| Iron Maiden - Number of the Beast |
| Ricky Martin - Livin’ la Vida Loca |
| Michael Bolton - When a Man Loves a Woman |
| Plain White Ts - Hey There Delilah |
| Flanders & Swann - The Gasman Cometh |
| Meatloaf - Bat Out of Hell |
| Oasis - (What's the Story) Morning Glory? |
| Landscape - Einstein a Go Go |
| Al Green - Let's Stay Together |
| Iron Maiden - The Number of the Beast |
| Bon Jovi - Slippery When Wet (album) |
| Mumford and Sons - Little Lion Man |
| Patsy Cline - Crazy |
| The Veronicas - Untouched |
| The Verve - Bittersweet Symphony |
| The Carpenters - Top of the World |
| Frank Turner - The Next Storm |
| Don MacLean - American Pie |
| 70s rock (radio station) |
| Queen - Who Wants to Live Forever |
| Madness - It Must Be Love |
| The Shakers - One Wonderful Moment |
| Queen - March of the Black Queen |
| Pink Floyd - Time |
| The Cure - The Lovecats |
| The Kooks |
| Sting - Fields of Gold |
| ABBA - Dancing Queen |
| Bob Segar - Still the Same |
| Yes - And You and I |
| Queens - We are the Champions |
| Madness - Greatest Hits (album) |
| Novo Amor - Carry You |
| Steeleye Span - Cam ye oer frae france |
| Phil Collins - In The Air Tonight |
| Genesis - Trick of The Tail |
| Kate Bush - Wuthering Heights |
| Culture Club - Karma Chameleon |
| Green Day - Basketcase |
| Madness - The Return of The Los Palmas 7 |
| Massy Star - Fade Into You |
| The Smiths |
| Madness - Wings of a Dove |
| Wave 105 (radio station) |
| Sinead O'Connor - Troy |
| Queen - Greatest Hits (album) |
| David Bowie - Scary Monsters and Super Creeps (album) |
| Snow Patrol - Chasing Cars; More Than a Favour - Jeff Jepson; Rachmaninov; Stardust Soundtrack; Pearl Jam - 10,000 maniacs; Chris Stapleton - Tennessee Whisky; Frank Sinatra |
| Foo fighters - Evermore |
| Luke Kelly - Scorn Not His Simplicity |
| Taylor Swift - Hey Stephen |
| Roberta Flack - First Time Ever I Saw Your Face |
| Evanescence - Don't Cry For Me |
| Boston - More Than a Feeling |
| Charles Trenet - La Mer |
| Fairport Convention - A Sailor's Life |
| Paul Young - Love of the Common People |
| Snow Patrol - Chasing Cars |
| John Lennon & Yoko Ono - Give Peace a Chance |
| Gustav Holst - Jupiter |
| Black Sabbath - Heaven and Hell |
| Carly Rae Jepsen - Call Me Maybe |
| Woolstone - The Chase; Queen; Mamma Mia the musical |
| Green Day - Basket case |
| Queen - Under Pressure |
| Mary Coughlan - Ancient Rain |
| Wonderful tonight. Eric clapton |
| Nothing comes to mind |
| The Beatles - Hey Jude |
| We'll Meet Again - Vera Lynn |
| Meatloaf - I would do anything for love (but I won't do that) |
| Rod Stewart - Mandolin Wind |
| Led Zeppelin - Stairway to Heaven |
| Austin Howard - Heaven Knows; Lana Del Rey - Blue Jeans |
| Joan Baez |
| The Shadows - Don't Make My Baby Blue |
| Adam and the Ants - Kings of the Wild Frontier |
| Strange Fruit - The Flame Still Burns |
| Simon & Garfunkel - Bridge Over Troubled Water |
| David Bowie - Hunky Dory (album) |
| Billy Fury - Halfway To Paradise |
| Eva Cassidy - Over The Rainbow |
| The Beatles - All You Need Is Love |
| Another Level - Bomb Diggy |
| Billy Joel - Tell Her About It |
| Corries - Dark Lochnagar |
| Monty Python - Always Look on the Bright Side of Life |
| Fleetwood Mac - Skies the Limit |
| More Than Words - Extreme |
| Blink 182 - All the Small Things |
| Sneaker Pimps - Six Underground |
| Peter Gabriel - In Your Eyes (live) |
| Elo - Ticket To The Moon |
| Genesis - Supper's Ready |
| Paul Simon - You Can Call Me Al |
| Bon Jovi - Just Older |
| Elgar - Cello concerto |
| R&B from the 2000s |
| The Fugees - Killing Me Softly |
| Sam Cooke |
| Whitesnake |
| Elbow - One Day Like This |
| Blur - Song 2 |
| Roxy Music - Avalon |
| Artemesia - Bits & Pieces |
| Queen - Under Pressure |
| The Damned - New Rose |
| Tiffany - I Think We're Alone Now |
| Suede |
| The Beatles - Things We Said Today |
| David Bowie - China Girl |
| Ennio Morricone - Chi Mai; Galloping Home (from The Adventures of Black Beauty); Various other themes from TV shows of the 70s and 80s |
| Cock Robin - The Promise You Make |
| Starships - Nothing's Gonna Stop Us Now |
| Manolo García - Nunca el Tiempo es Perdido |
| Calon Lân (Welsh Traditional) |
| Belly - Judas My Heart |
| Sonique - It Feels So Good |
| Harry Nilsson - Without You |
| Hazel O'Connor - Will You |
| The Andrews Sisters - Beer Barrel Polka |
| Underworld - Born Slippy |
| Elgar - Enigma Variations |
| 4 Non-Blondes - What's up |
| Paul Simon - Homeward Bound |
| Geoff Wain - Forever Autumn |
| Jim Reeves |
| Tracy Chapman - Fast Car |
| Blink 182 - I'm Lost Without You |
| Van Morrison |
| Paul Simon - Graceland (album) |
| Tallis - If Ye Love Me |
| Xiaoou Hu - I Must Come Back |
| Bloc Party - Flux |
| The O'Jays - Backstabbers |
| Freya Ridings - Lost Without You - |
| Wild Theme from Local Hero (Mark Knopfler) |
| I'm On My Way (Dangdut) |
| Beyonce |
| Muse - Unintended |
| A Tribe Called Quest - Midnight Marauders |
| Armistice - Phoenix; Passion Pit - The Reeling |
| Baz Luhrmann - Sunscreen |
