# Supplementary material for: Music-Evoked Nostalgia and Wellbeing During the United Kingdom COVID-19 Pandemic: Content, Subjective Effects, and Function
Source: Front Psychol. 2021 Mar 22;12:647891. doi: 10.3389/fpsyg.2021.647891 (PMC8019926; doi:10.3389/fpsyg.2021.647891)
Supplement: Supplementary file 4 [file Table_4.DOCX]

S4: Tukey Post Hoc Comparisons (Fig. 1 and Fig. 3)

| Table A. Fig. 1 Tukey Post hoc paired comparisons for *EEN* items (outcome variables) and coded valence groups (predictor variables) | | | | | | | |
| --- | --- | --- | --- | --- | --- | --- | --- |
| **EEN *Happy Past Times*** (F(557,3)=21.456, *p*<.001, Adjusted *R^2^*=.099, *η2*=.104) | | | | | | | |
| Coded Valence Group | | | Mean Difference  (1-2) | *SE* | *p* | 95% Confidence Interval | |
| 1 | 2 | |  |  |  | Lower Bound | Upper Bound |
| Positive | | Negative | 1.320*** | 0.187 | <.001 | 0.835 | 1.800 |
| Positive | | Mixed | 0.420* | 0.153 | .032 | 0.026 | 0.813 |
| Positive | | Neutral | 1.530*** | 0.367 | <.001 | 0.588 | 2.478 |
| Negative | | Mixed | -0.900*** | 0.219 | <.001 | -1.463 | -0.334 |
| Mixed | | Neutral | 1.110* | 0.384 | 0.020 | 0.124 | 2.104 |
| ***EEN Sad Past Times*** (F(557,3)=33.106, *p*<.001, Adjusted *R^2^*=.147, *η2*=.151) | | | | | | | |
| Coded Valence Group | | | Mean Difference  (1-2) | *SE* | *p* | 95% Confidence Interval | |
| 1 | 2 | |  |  |  | Lower Bound | Upper Bound |
| Positive | | Negative | -2.130*** | 0.245 | <.001 | -2.757 | -1.495 |
| Positive | | Mixed | -1.240*** | 0.200 | <.001 | -1.755 | -0.725 |
| Negative | | Mixed | 0.890* | 0.287 | 0.011 | 0.148 | 1.625 |
| Negative | | Neutral | 2.280*** | 0.522 | <.001 | 0.936 | 3.628 |
| Mixed | | Neutral | 1.400* | 0.503 | 0.029 | 0.100 | 2.691 |
| ***EEN Appreciation*** (F(557,3)=12.066, *p*<.001, Adjusted *R^2^*=.056, *η2*=.061) | | | | | | | |
| Coded Valence Group | | | Mean Difference  (1-2) | *SE* | *p* | 95% Confidence Interval | |
| 1 | 2 | |  |  |  | Lower Bound | Upper Bound |
| Positive | | Negative | 0.730** | 0.221 | 0.006 | 0.156 | 1.294 |
| Positive | | Mixed | 0.760*** | 0.180 | <.001 | 0.292 | 1.221 |
| Positive | | Neutral | 1.730*** | 0.433 | <.001 | 0.610 | 2.841 |
| ***EEN Sad Difficult Times*** (F(557,3)=24.294, *p*<.001, Adjusted *R^2^*=.111, *η2*=.116) | | | | | | | |
| Coded Valence Group | | | Mean Difference  (1-2) | *SE* | *p* | 95% Confidence Interval | |
| 1 | 2 | |  |  |  | Lower Bound | Upper Bound |
| Positive | | Negative | -1.940*** | 0.243 | <.001 | -2.564 | -1.310 |
| Positive | | Mixed | -0.860*** | 0.198 | <.001 | -1.375 | -0.353 |
| Negative | | Mixed | 1.070** | 0.285 | .001 | 0.339 | 1.806 |
| Negative | | Neutral | 1.930** | 0.518 | .001 | 0.592 | 3.264 |
| ***EEN Bittersweet*** (F(557,3)=32.476, *p*<.001, Adjusted *R^2^*=.144, *η2*=.149) | | | | | | | |
| Coded Valence Group | | | Mean Difference  (1-2) | *SE* | *p* | 95% Confidence Interval | |
| 1 | 2 | |  |  |  | Lower Bound | Upper Bound |
| Positive | | Negative | -1.680*** | 0.235 | <.001 | -2.284 | -1.072 |
| Positive | | Mixed | -1.470*** | 0.192 | <.001 | -1.968 | -0.979 |
| Neutral | | Negative | -2.160*** | 0.502 | <.001 | -3.451 | -0.867 |
| Mixed | | Mixed | -1.950*** | 0.483 | <.001 | -3.198 | -0.711 |
| *Note*: **p*<.05, ***p*<.01,****p*<.001, $\boldsymbol{\dagger}$non-significant trend at *p*<.10, *EEN Reminder of People* item not included in this table, as no significant comparisons were observed, *n*=561. | | | | | | | |

| Table B. Fig 3. Tukey Post hoc paired comparisons for *ERS-ACA* factor scores (outcome variables) and coded valence groups (predictor variables) | | | | | | | |
| --- | --- | --- | --- | --- | --- | --- | --- |
| **Avoidance Factor Score** (F(557,3)=10.553, *p*<.001, Adjusted *R^2^*=.049, *η2*=.054) | | | | | | | |
| Coded Valence Group | | | Mean Difference  (1-2) | *SE* | *p* | *95% Confidence Interval* | |
| 1 | 2 | |  |  |  | *Lower Bound* | *Upper Bound* |
| Positive | | Negative | .246 $\boldsymbol{\dagger}$ | .096 | .053 | -.002 | .493 |
| Positive | | Mixed | .331*** | .078 | .000 | .129 | .533 |
| Positive | | Neutral | .700** | .188 | .001 | .215 | 1.185 |
| **Approach Factor Score** (F(557,3)=5.773, *p*=.001, Adjusted *R^2^*=.025, *η2*=.030) | | | | | | | |
| Coded Valence Group | | | Mean Difference  (1-2) | *SE* | *p* | *95% Confidence Interval* | |
| 1 | 2 | |  |  |  | *Lower Bound* | *Upper Bound* |
| Neutral | | Positive | -.588** | .154 | <.001 | -.985 | -.191 |
| Neutral | | Negative | -.495* | .168 | .017 | -.928 | -.063 |
| Neutral | | Mixed | -.467* | .161 | .020 | -.883 | -.051 |
| **Self-Development Factor Score** (F(557,3)=6.878, *p*=.001, Adjusted *R^2^*=.031, *η2*=.036) | | | | | | | |
| Coded Valence Group | | | Mean Difference  (1-2) | *SE* | *p* | *95% Confidence Interval* | |
| 1 | 2 | |  |  |  | *Lower Bound* | *Lower Bound* |
| Positive | | Negative | .233 $\boldsymbol{\dagger}$ | .093 | .062 | -.008 | .473 |
| Neutral | | Positive | -.691** | .183 | .001 | -1.163 | -.220 |
| Neutral | | Negative | -.459 $\boldsymbol{\dagger}$ | .199 | .099 | -.972 | .055 |
| Neutral | | Mixed | -.544* | .192 | .024 | -1.038 | -.050 |
| *Note*: **p*<.05, ***p*<.01,****p*<.001, $\boldsymbol{\dagger}$non-significant trend at *p*<.10, *n*=561. | | | | | | | |
