# Supplementary material for: Music-Evoked Nostalgia and Wellbeing During the United Kingdom COVID-19 Pandemic: Content, Subjective Effects, and Function
Source: Front Psychol. 2021 Mar 22;12:647891. doi: 10.3389/fpsyg.2021.647891 (PMC8019926; doi:10.3389/fpsyg.2021.647891)
Supplement: Supplementary file 5 [file Table_5.DOC]

S5: Coded Reasoning and Emotion Regulation Strategies
